# Identification of SIRT3 as an eraser of H4K16la

**DOI:** 10.1016/j.isci.2023.107757

**Published:** 2023-08-28

**Authors:** Zhuming Fan, Zhiyang Liu, Nan Zhang, Wenyu Wei, Ke Cheng, Hongyan Sun, Quan Hao

**Affiliations:** 1 Institute of High Energy Physics, CAS, Beijing 100000, China; 2 Spallation Neutron Source Science Center, CAS, Dongguan, Guangdong 523000, China; 3 School of Biomedical Sciences, The University of Hong Kong, Hong Kong, China; 4 Department of Chemistry and COSDAF (Centre of Super-Diamond and Advanced Films), City University of Hong Kong, Hong Kong, China

**Keywords:** Enzymology, Protein, Molecular interaction, Structural biology

## Abstract

Lysine lactylation (Kla) is a novel histone post-translational modification discovered in late 2019. Later, HDAC1-3, were identified as the robust Kla erasers. While the Sirtuin family proteins showed weak eraser activities toward Kla, as reported. However, the catalytic mechanisms and physiological functions of HDACs and Sirtuins are not identical. In this study, we observed that SIRT3 exhibits a higher eraser activity against the H4K16la site than the other human Sirtuins. Crystal structures revealed the detailed binding mechanisms between lactyl-lysine peptides and SIRT3. Furthermore, a chemical probe, p-H4K16laAlk, was developed to capture potential Kla erasers from cell lysates. SIRT3 was captured by this probe and detected via proteomic analysis. And another chemical probe, p-H4K16la-NBD, was developed to detect the eraser-Kla delactylation processes directly via fluorescence indication. Our findings and chemical probes provide new directions for further investigating Kla and its roles in gene transcription regulation.

## Highlights

• SIRT3 shows a preference for delactylating H4K16 site compared to other Sirtuins
• Crystal structures revealed the binding details between lactyl-motif and SIRT3
• The probe with clickable photo-cross-linker captured SIRT3 from cell lysates
• The fluorogenic probe can be used to monitor the delactylation processes directly

## Graphical abstract

**Figure:**
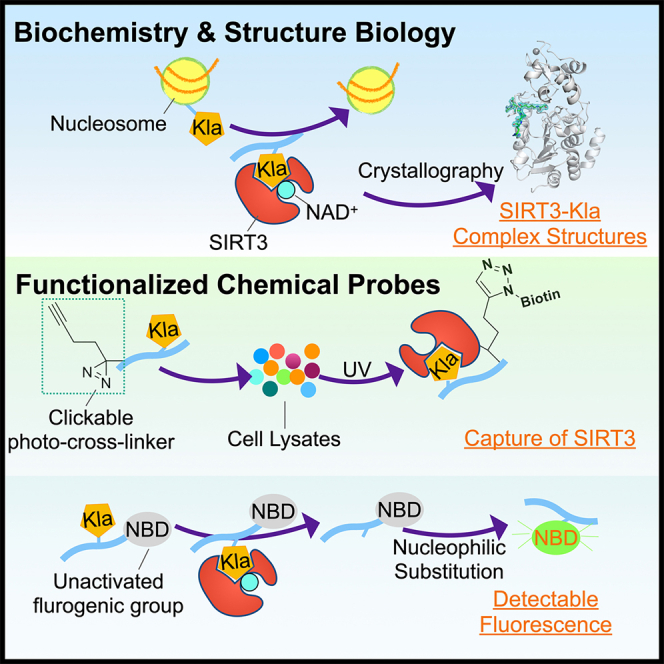

## Introduction

The fundamental subunit of eukaryotic chromatin is the nucleosome. It is composed of an octamer of 8 histone proteins, including 2 copies of H2A, H2B, H3, and H4, wrapped around by a 147 base-pair duplex DNA. The highly packaged and condensed chromatin formed by repeated nucleosome units represses transcriptional activity.^1^ However, histone post-translational modifications (PTMs) reveal new insights into gene transcription regulation. With the development of mass spectrometry-based proteomic analysis, numerous types and sites of histone PTMs have been discovered, mainly occurring at the N- and C-terminal tail of histones. Dynamic changes in histone modifications create temporary opportunities for nucleosome-DNA unwrapping and rewrapping, exposing specific sections of DNA for protein binding, resulting in the activation or suppression of gene transcription.^2^^,^^3^

Lactate, the end product of anaerobic glycolysis, replenishes 2 ATP per glucose without oxygen consumption. In 2019, it was discovered that lactate is not only a metabolite but also a signaling molecule. Zhang et al.^4^ reported lysine lactylation as a novel histone PTM that directly stimulates gene transcription similar to histone acetylation. Mass spectrometry analysis identified 26 histone lactyl-lysine sites in human HeLa cells. Additionally, ^13^C isotopic labeling techniques confirmed that lactylation levels are highly sensitive to lactate production from glycolysis. Notably, the accumulation of lactylation at the H3K18 site acts as a “lactate timer,” stimulating the transcription of M2-macrophage polarization genes and activating M2-related wound healing pathways to regulate macrophage polarization. The discovery of histone lactylation is a significant milestone toward understanding histone dynamics.

Histone deacetylases (HDACs), including HDACs and Sirtuin family proteins, are well studied PTM erasers that regulate gene transcription. Recently, Moreno-Yruela et al. discovered that class I HDACs (HDAC1-3), especially HDAC3, are effective lactylation erasers.^5^ They found that the *in vitro* enzymatic delactylation activity of HDAC1-3 is higher than that of Sirtuin family proteins. The authors also treated cells with HDAC inhibitors (butyrate, trichostatin A, and apicidin) and a Sirtuin pan-inhibitor (nicotinamide) to compare the intracellular delactylation activities between HDACs and Sirtuins. The results showed that class I HDACs were the major cellular delactylases because the nicotinamide treatment was unable to induce Sirtuin-regulated pan-Kla increasement, however, the pan-Kac level was also unchanged after treatment with nicotinamide.^5^ The catalytic mechanisms and physiological functions of HDACs and Sirtuins are different: HDACs require tandem histidine residues to serve as general base catalysts for deprotonating the zinc-bond water to activate it to attack the carbonyl group of acyl-lysine^6^; on the other hand, Sirtuins catalyze deacylation reactions but require NAD^+^ (Nicotinamide adenine dinucleotide) molecules for the reaction. Sirtuins highly sense changes in intracellular NAD^+^ levels and regulate gene transcription by modulating protein acylation.^7^

Emerging biological and structural evidence suggests that Sirtuins are not limited to erasing acetyl groups from lysine residues but can also remove other forms of acyl groups. Sirtuins can erase a range of acyl-lysine groups, from short acylation with a 2-carbon backbone to long-chain fatty acylation with a 16-carbon backbone.^8^ The zinc-binding domain and Rossmann fold domain make up the highly conserved catalytic core of Sirtuins.^9^^,^^10^ During the enzymatic reaction, the acyl-lysine first binds to the active site of the Sirtuin enzyme, followed by the binding of NAD^+^. This binding triggers a series of nucleophilic substitution reactions that catalyze the hydrolyzation of the modification and the formation of a nicotinamide molecule and O’acyl-ADP-ribose.^11^^,^^12^ Researches indicate that SIRT1-3 effectively remove fatty-acyl modifications such as acetyl-lysine (Kac, 2 carbons) and propionyl-lysine (Kpr, 3 carbons).^13^^,^^14^^,^^15^^,^^16^ In comparison, enzymatic studies demonstrated that SIRT4 acts as a cellular lipoamidase.^17^ The human SIRT4 structure hasn’t been solved yet, but a *X. tropicalis* SIRT4 structure revealed the dehydroxymethylglutarylation activity of SIRT4.^18^ Whereas SIRT5 binds strongly to modifications containing the carboxyl tail, such as succinyl-lysine (Ksuc) and glutaryl-lysine (Kglu).^19^^,^^20^ SIRT6 functions best against long-chain fatty acyl-lysine, such as myristoyl-lysine (Kmyr) and palmitoyl-lysine (Kpalm).^21^ Conversely, aside from the de-fatty-acyl-lysine function, SIRT3 shows a higher affinity for removing modifications that include hydrophilic hydroxyl-motifs, such as β-hydroxybutyryl-lysine (Kbhb) and 2-hydroxyisobutyryl-lysine (Khib).^22^ Since lactyl-lysine also has similar hydroxyl-motif, we hypothesize that SIRT3 may potentially display higher delactylation activity compared to other human Sirtuins.

To determine the delactylation activity of Sirtuins, we conducted various biochemical and cellular experiments. Our findings indicate that SIRT3 exhibits higher delactylation activity compared to other human Sirtuins, especially on H4K16 site. Crystal structures showed that SIRT3 interacts with Kla-peptides through a preconfigured hydrophobic pocket that accommodates the hydrocarbon portion of the lactyl-lysine. The interaction between SIRT3 and the hydrophilic part of the lactyl motif is facilitated by a hydrogen bond network that incorporates water molecules. Furthermore, we developed a functionalized chemical probe with a clickable photo-cross-linker, p-H4K16laAlk probe, that successfully captured SIRT3 from cell lysates. A fluorogenic probe, p-H4K16la-NBD, was also developed to directly detect Kla-eraser delactylation processes via fluorescence indication. Our findings and newly developed probes support previous Kla eraser studies and open new avenues for further research in the field.

## Results

### SIRT3 shows higher delactylation activity on H4K16 site compared to other human Sirtuins

With NAD^+^ consumption/cycling assay, lactylated H3K9 (identified in human HeLa cells), H3K14 (identified in mouse BMDM cells), H3K56 (identified in mouse BMDM cells) and H4K16 (identified in human HeLa cells) sites were chosen, and lactyl peptides were purchased for the preliminary screening as these sites are Sirtuins’ “preferred sites.” These four sites have been widely reported that different modifications on these sites can be erased by Sirtuins. And many co-crystal structures of these acyl-lysines and Sirtuins have been solved (Table S1). Due to SIRT4’s demethylglutarylase- and deslipoylasehas-f activity and inability to express in soluble form in *E. coli*, there is a limited yield of SIRT4 protein during purification.^19^ Therefore, we first prepared all truncated human Sirtuins based on PDB-published Sirtuin-ligand structure sequences except for SIRT4 for the following enzymatic assays and crystallographic study (Figure S1).

**Figure 1:**
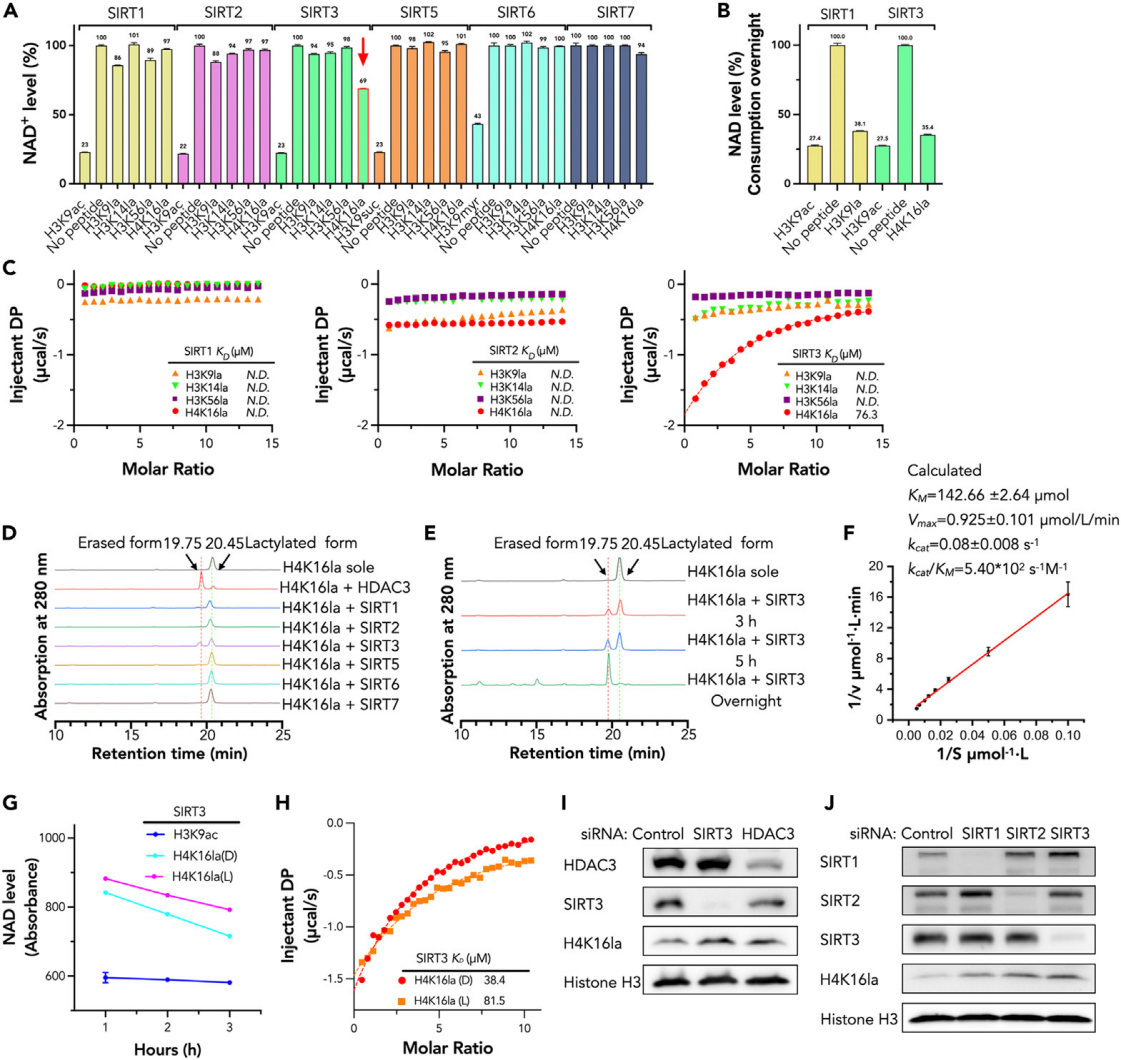
SIRT3 shows higher delactylation on H4K16la compared to other human Sirtuins (A) 3 h NAD^+^ consumption/cycling assay of SIRT1, 2, 3, 5, 6, 7 on H3K9la, H3K14la, H3K56la and H4K16la peptides. (B) Overnight consumption/cycling assay of SIRT1 and SIRT3 on H3K9la and H4K16la, respectively. (C) ITC fitting curves of SIRT1-3 titrated with H3K9la, H3K14la, H3K56la and H4K16la. (D) HPLC-comparison of the H4K16la erasing capacity between Sirtuins, and HDAC3 was set as the positive control (3 h incubation). (E) HPLC-time-dependent cleavage assay of SIRT3 with H4K16la peptide. (F) The fitted curve of delactylation speed (v) and original concentration of H4K16 peptide ([S]). Based on the equation, $\frac{1}{v}=\left(\frac{K_{M}}{V_{\max}}\right)\left(\frac{1}{\left[S\right]}\right)+\frac{1}{V_{\max}}$, calculated *K*_*M*_ = 142.66 ± 2.64 μM, *V*_*max*_ = 0.925 ± 0.101 μmol/L/min, *k*_*cat*_ = 0.08 ± 0.008 s^−1^, *k*_*cat*_/*K*_*M*_ = 5.40x10^2^ s^−1^M^−1^. (G) NAD^+^ consumption/cycling assay of SIRT3 with H4K16la(D/L) peptide. (H) ITC fitting curves of SIRT3 titrated by H4K16la(D/L) peptides. (I) Western blot analysis of cellular H4K16la level change after 48 h post-knockdown of SIRT3 and HDAC3 via siRNA in HEK293T cells. (J) Western blot analysis of cellular H4K16la level change after 48 h post knockdown of SIRT1-3 via siRNA in HEK293T cells.

In comparison to the negative control group (no peptide), the SIRT5-7 groups exhibited minimal changes in NAD^+^ levels. While the class I Sirtuins, SIRT1-3, as previous reported, showed a certain delactylation activities against lactyl peptides. Among the tested combination of Sirtuins and lactyl peptides, SIRT3 showed a higher delactylation activity on H4K16la and H3K23la peptide. (Figure 1A; Figures S2A and S2B). Extending the incubation time to overnight (≥12 h), both SIRT1-H3K9la and SIRT3-H4K16la consumed much higher level of NAD^+^ (Figure 1B). Next, we performed ITC (Isothermal titration calorimetry) to compare the binding affinity between SIRT1-3 with H3K9la, H3K14la, H3K56la, and H4K16la peptides. Consistent with the NAD^+^ cycling assay result, the binding affinity of SIRT3-H4K16la measured (76.3 μM) is much higher than SIRT1-2 with these lactyl peptides (Figure 1C; Figures S3–S5).

We also performed HPLC-MS analysis to verify this result. Results showed that in comparison with Sirtuins, the reported robust Kla eraser, HDAC3, showed the highest *in vitro* delactylation activity against H4K16la. While SIRT3 exhibited the highest erased ratio against H4K16la peptides compared with other Sirtuins (Figures 1D and 1E, and the mass results showed in Figure S6). More peptides were erased by SIRT3 after 3 h of incubation, while SIRT1 only showed slight hydrolytic activity against H3K9la and H4K16la. Additionally, those Sirtuins didn’t show any hydrolytic activity against H4K12la peptides (Figure S7). Furthermore, we studied the kinetics of SIRT3-H4K16la by measuring the *K*_*M*_ and *k*_*cat*_ values via HPLC method. The fitted curve calculated *K*_*M*_ = 142.66 ± 2.64 μM, *V*_*max*_ = 0.925 ± 0.101 μmol/L/min, *k*_*cat*_ = 0.08 ± 0.008 s^−1^, *k*_*cat*_/*K*_*M*_ = 5.40∗10^2^ s^−1^M^−1^ (Figure 1F; Figure S8). Based on these findings, it seems that the *in vitro* delactylation activity of SIRT3 on the H4K16 site is comparable in magnitude to its de-β-hydroxybutyrylation activity on H4K16, as indicated by a *k*_*cat*_/*K*_*M*_ value of 135.09 s^−1^M^−1^ reported by Zhang et al.^22^ However, HDAC3 exhibit significantly higher *in vitro* delactylation activity, as indicated by the *k*_*cat*_/*K*_*M*_ value on lactyl peptides (＞4000 s^−1^M^−1^).^5^^,^^23^ Collectively, these results verified the delactylation activity of SIRT3 on H4K16 site.

### Binding priority of SIRT3 to D- and L- H4K16la

Lactyl-lysine has structural similarities with other acyl-lysines, such as propionyl-lysine, 2-hydroxyisobutyryl-lysine, and β-hydroxybutyryl-lysine, has a unique chiral center at the 3-carbon-site. Compared to the R-configure Kbhb, SIRT3 exhibits higher binding affinity with the S-configure Kbhb. We investigated whether SIRT3’s delactylation activity showed differences on lactylated L- and D-lysine peptides by performing the NAD^+^ consumption/cycling assay and ITC experiments. Results showed that SIRT3 preferred hydrolyzing lactylated D-lysine over lactylated L-lysine, as it consumed more NAD^+^ after incubation with the former. The binding affinity of SIRT3 to lactylated D-lysine peptide was also found to be approximately 2-fold higher than that of lactylated L-lysine peptide. (Figures 1G and 1H; Figure S9). These results proved SIRT3 also preferred to hydrolyze lactylated D-lysine other than lactylated L-lysine.

In vertebrate metabolism, L-LDH plays a crucial role in the conversion of lactate from pyruvate, and the lactate produced from glycolysis is almost exclusively in L-configuration.^24^ D-lactate, on the other hand, can only be absorbed from exogenous sources or be converted by very few D-LDH.^25^ Although both HDACs and SIRT3 showed a preference for hydrolyzing lactylated D-lysine, further investigation is necessary to fully understand the physiological existence and function of D-lactylation. This may include exploring the biological processes and pathways that are regulated by D-lactylation, as well as identifying the enzymes involved in D-lactylation and their catalytic mechanisms.

### SIRT3 knockdown affects H4K16la level in living cells

Then, we investigated if SIRT3 can regulate intracellular lactylation levels. Cellular localizations of seven human Sirtuins are different. SIRT6 and SIRT7 only exist in nuclei. SIRT1 mainly locates at nuclei but also exists in cytoplasm,^26^ while SIRT2 is primarily located at the cytoplasm but also exists in nuclei. SIRT3, SIRT4, and SIRT5 are regarded as the mitochondrial Sirtuins.^27^ However, more evidence (listed in Table S2) shows that SIRT3 also exists in a nuclei and acts as the histone deacylase.

To investigate SIRT3’s delactylation activity on physiological nuclear substrates in living cells, we performed a siRNA knockdown assay in living HEK293T cells. Knocking down either SIRT3 or HDAC3 increased cellular H4K16la level (Figure 1I; Figure S10). Moreover, knocking down SIRT3 resulted in a more significant enhancement of H4K16la levels compared to the knockdown of SIRT1/2 (Figure 1J; Figure S11). These data supported the nuclear localization of SIRT3, acting as a nuclear delactylase.

### Structural basis of SIRT3 with lysine lactylation peptides

Next, we performed crystallographic studies to gain insight into the molecular basis of SIRT3’s interaction with lactyl peptides. Crystals of H3K23la(L)-SIRT3 and H4K16la(L)-SIRT3 complexes diffracted to 2.0 Å and 2.5 Å resolution, respectively. A structural model from the PDB: 3GLS, was employed as the search model of molecular replacement. The SIRT3-H3K23la(L) and SIRT3-H4K16la(L) complex structures were well-built and refined (data collection and refinement statistics are displayed in Table S3).

**Figure 2:**
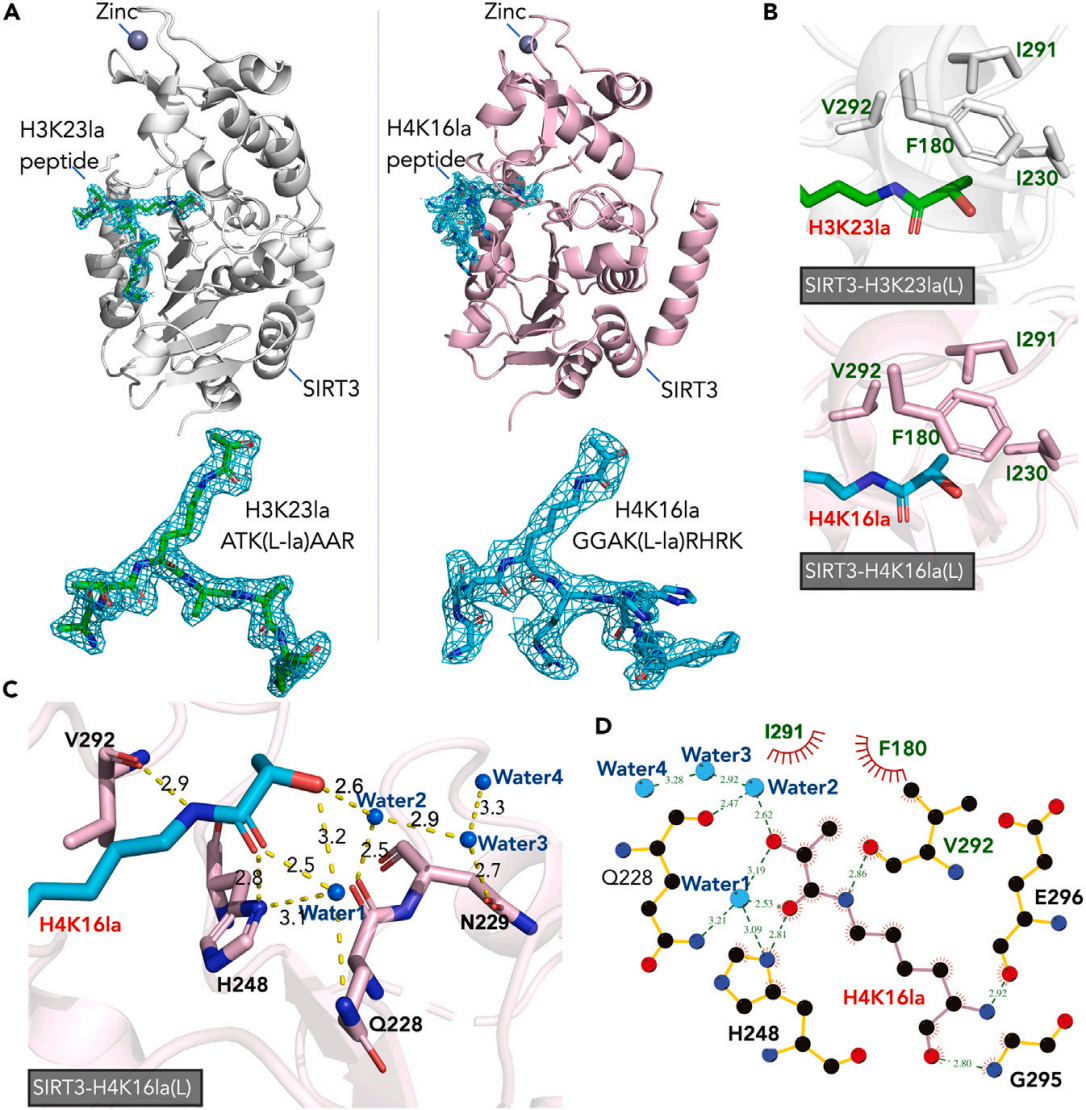
The structural basis of SIRT3 and lactyl-lysine peptides (A) Left: The overall structures of SIRT3(white)-H3K23la(L)(green) complex structure (white) and the ligand models observed in the SIRT3-H3K23la(L) structure. Right: The overall structures of SIRT3(light pink)-H4K16la(L)(cyan) complex structure and the ligand models observed in the SIRT3-H4K16la(L) structure. The 2Fo-Fc maps displayed for the ligands are rendered at 0.9σ contour. (B) The preconfigured hydrophobic cage of SIRT3 accommodates the hydrocarbon portion of the lactyl group. Upper: SIRT3(white)-H3K23la(L)(green). Lower: SIRT3(light pink)-H4K16la(L)(cyan). (C) Ligand-protein interaction details in SIRT3(light pink)-H4K16la(L)(cyan) structure. Hydrogen bond is represented as a yellow dashed line, and the water molecule is represented as spheres (marine). The length (Å) of the hydrogen bond is labeled next to the dashed line. (D) LIGPLOT diagram list interactions between the H4K16(L) ligand and SIRT3. H4K16la(L) (bond color: light pink) and residues of SIRT3 (bond color: gold) are depicted in ball-and-stick mode. Carbon is represented as a black ball; nitrogen is represented as a blue ball; oxygen is represented as a red ball; water molecule is represented as a marine ball; hydrophobic interactions are shown as red arcs; and hydrogen bond is represented as a green dashed line. The length (Å) of the hydrogen bond is labeled next to the dashed line.

Each structure contains 1 SIRT3 molecule, 1 zinc atom, and 1 lactyl peptide in each asymmetric unit. The 2Fo-Fc map clearly captured more than 6 amino acids’ electron density (Figure 2A). The catalytic pocket of SIRT3 displayed half-hydrophobic and half-hydrophilic characters. In both structures, F180, I230, I291, and V292 of SIRT3 formed a preconfigured hydrophobic cage that accommodated the hydrocarbon portion of the lactyl group (Figure 2B). Similar to other published SIRT3-acyllysine-peptide complex structures (PDB: 5BWN, SIRT3 with Kmyr peptide, and PDB: 5Z93, SIRT3 with Kbhb peptide, show in Figure S12), the amido linkage in SIRT3-H4K16la(L) was stabilized by V292, H248, and Q228 though direct hydrogen bonds as well as water bridges (Figure 2C). Several water molecules participated in the hydrogen bond network to facilitate the interaction between the hydroxyl group of the Kla and Q228, N229, H248 of SIRT3 in SIRT3-H4K16la(L) structure (Figures 2C and 2D). While in the SIRT3-H3K23la(L) structure, due to the crystallization conditions involving glycerol, two glycerol molecules’ hydroxyl groups replaced the water molecule’s position and contributed to the formation of the water bridge, resulting in a robust hydrogen bond system (Figure S13). Collectively, these structural data supported SIRT3’s ability to accommodate lactyl-lysine and carry out the subsequent NAD^+^-consumption-based catalytic reaction.

### Development of the functionalized chemical probes

**Figure 3:**
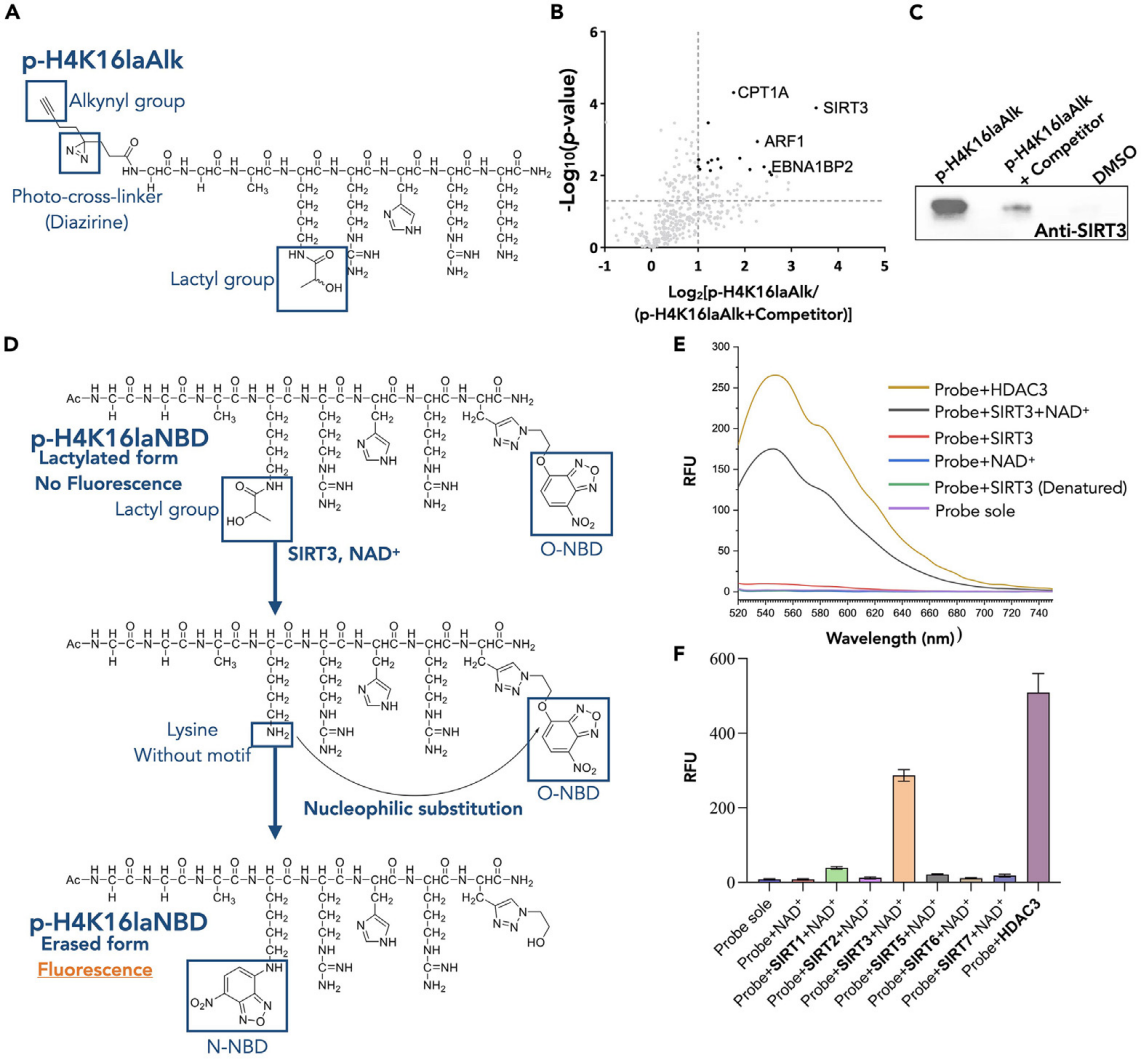
Development of the chemical probes to investigate lysine lactylation (A) Chemical structure of the p-H4K16laAlk. (B) Proteomic analysis results of p-H4K16laAlk pull down assay (the dotted lines represent p = 0.05 and the enriched ratio = 2). (C) Western blot analysis of detecting the existence of SIRT3 in the pull-down products of group_p-H4K16laAlk,_ group_p-H4K16laAlk with competitor_ and DMSO group (negative control). (D) Chemical structure of the fluorogenic probe, p-H4K16laNBD and the schematic diagram of the reaction mechanism. (E) Fluorescence spectrum of p-H4K16laNBD probe (λex = 480 nm). Probe: 10 μM, SIRT3: 0.2 μM, HDAC3: 0.1 μM, and NAD^+^: 100 μM. (F) The Fluorescence of p-H4K16laNBD (10 μM) with different enzymes (enzyme concentration: 0.1 μM; λex = 480 nm; λem = 545 nm). Enzymatic reaction condition: enzymes (0.1 μM) in 20 mM HEPES buffer (pH = 8.0) containing 100 μM NAD^+^ at 37°C for 2 h. (No NAD^+^ was added in HDAC3 group).

Chemical affinity-based probes are powerful tools that convert weak protein-ligand interactions into robust covalent chemical linkages, thus enabling the capture of transient interactions. To validate the delactylation activity of endogenous SIRT3 and profile the transient interactions between other potential erasers/readers and Kla substrates, we first generated a lactate mimic with clickable alkynyl group (Figure S14), in which the alkynyl group could be subsequent biorthogonal conjugated to a biotin tag for the following streptavidin pull-down and proteomic analysis. We tried to use this lactate mimic to induce the increasement of pan-alkynyl-lactylation level. It has been reported that the treatment of sodium lactate on cells for 24 h will induce detectable levels of lysine lactylation.^4^ While our lactate mimic showed unavoidable cytotoxicity against MCF-7 cells and HeLa cells. Therefore, based on our biochemistry data and previous experience with PTM probes, we designed and synthesized a H4K16la based tagging system that covalently links a H4K16la peptide (p-H4K16laAlk) (Figure 3A). The p-H4K16laAlk consists of 3 parts, i.e., ①the affinity-based part: peptide with H4K16Kla sequence and modification for target protein recognition. ②A photo-cross-linker (i.e., diazirine) at the N-terminus of the peptide, which could convert the transient interactions between peptide ligand and proteins into robust and irreversible covalent bonds after ultra-violet (UV) irradiation. ③ An alkynyl group for biotin clicking. This strategy has been widely employed to identify potential binding targets for molecules of interest.^28^^,^^29^^,^^30^

We performed the UV induced photo-cross-linking in 2 groups, group_p-H4K16laAlk_ and group_p-H4K16laAlk with competitor (H4K16la peptide)_ in U251 cell lysates. And after click chemistry reaction, the proteins bound in these two groups were pulled down by streptavidin agarose beads and sent for HPLC-MS/MS analysis. After proteomics analysis, we identified 56 proteins that preferentially enriched in group_p-H4K16laAlk_ compared with the group_p-H4K16laAlk with competitor_. And SIRT3 was identified successfully with enriched ratio ≥2 and with p value ≤ 0.05 (Figure 3B; Table S4). The preferentially enrichment of SIRT3 by p-H4K16laAlk was further validated by Western blot assay (Figure 3C; Figure S15). Hence, the probe p-H4K16laAlk validated that H4K16la is the substrate of SIRT3 in cellualr environment.

Additionally, another H4K16la based fluorogenic probe, p-H4K16laNBD, was developed to detect the delactylation process directly. This probe contains the H4K16la sequence and lactylation modification, as well as an O-NBD (Nitrobenzofurazan dye) group that is conjugated to the C-terminus. When the lactyl group is erased by the related enzymes, the exposed amino group can undergo an attack on the O-NBD moiety, resulting in its conversion to an N-NBD group and generating a strong fluorescence that can be easily detected (Figure 3D). Based on the fluorescence generated, we can compare the delactylation activities of various enzymes and calculate the delactylation reaction constant.

The p-H4K16laNBD showed an absorption peak at around 370 nm. After the 10 μM probe was incubated with 0.2 μM SIRT3 and 100 μM NAD^+^ for 2 h, we observed lower peak at 370 nm and a new peak at 482 nm, the generated N-NBD product was verified by LC-MS (Figures S16A and S16B). Further, the fluorescence spectrum was tested under 480 nm excitation (Figure 3E). We used HDAC3 as the positive control. From the results, the probe showed similar fluorescence spectrum with an emission peak at 550 nm after incubating with SIRT3 and NAD^+^. The other groups showed that sole NAD^+^ or denatured SIRT3 will not affect the fluorescence generating. These results verified the ability of this probe for screening delactylation activity on H4K16 site. Using this probe to screen the delactalytion activities of other Sirtuins (Figure 3F), the results showed that HDAC3 and SIRT3 groups fluoresced at 550 nm while other Sirtuins displayed minimal changes in fluorescence (Figure 3F). We also conduct time-dependent fluorescence measurement to calculate the reaction constant using this probe. And related constants are 0.0102 min^−1^ for HDAC3, and 0.0059 min^−1^ for SIRT3 (Figure S16C). Together, these results showed this fluorogenic probe is suitable for the screening of delactylation proteins and measurement of reaction rate for delactylation processes.

## Discussion

In this study, our results demonstrate that SIRT3 exhibits the highest activity against the H4K16la site compared to other Sirtuins. Through the complex structures of SIRT3 with lactyl peptides, we have elucidated the binding mechanisms between SIRT3 and lactyl peptides. Our findings reveal that SIRT3 possesses a preconfigured hydrophobic pocket that specifically accommodates the hydrocarbon part of lactyl-lysine. Furthermore, a water bridge network involving Q228, N229, and H248 of SIRT3 stabilizes the hydroxyl group of lactyl-lysine. Notably, the clickable photo-cross-linker probe, p-H4K16laAlk, together with proteomic analysis, have identified and verified SIRT3 as the binder of the H4K16la substrate. Additionally, the fluorogenic probe, p-H4K16laNBD, was able to detect delactylation activity directly, making it possible to screen the delactylation activities of various enzymes. The findings and newly designed chemical probes of this study could have a significant impact on the field of SIRT3-related Kla regulation, and further research into this area is warranted.

### Limitations of study

The catalytic mechanisms of Sirtuins and HDACs differ, implying potential variations in their physiological functions in relation to delactylation activities. In addition to the intracellular knockdown assay performed in this study, it is desirable to conduct experiments such as RNA-sequencing and Chip-sequencing to further understand the distinct gene transcription regulation functions of HDACs and Sirtuins.

## STAR★Methods

### Key resources table

**Table:** 

REAGENT or RESOURCE	SOURCE	IDENTIFIER
**Antibodies**		

Anti-H4K16la	PTM Biolabs	PTM-1417
Anti-Histone H3	Abclonal	A2348
Anti-SIRT1	Abclonal	A19667
Anti-SIRT2	Abclonal	A0273
Anti-HDAC3	Abclonal	A2139
Anti-SIRT3	Cell Signaling Technology	5490

**Bacterial and virus strains**		

BL21-CodonPlus (DE3)-RIL	Agilent Technologies	230245

**Chemicals, peptides, and recombinant proteins**		

Recombinant HDAC3/NCOR2	Abcam	ab42631
Lactyl peptides used in NAD^+^ consumption/cycling assay and ITC	SciLight Biotechnology	N/A
Lactyl peptides used in HPLC/MS	Synpeptide Co., Ltd.	N/A

**Deposited data**		

Proteomic data analysis	This paper	PXD039731
SIRT3-H3K23la structure	This paper	PDB: 8HLY
SIRT3-H4K16la structure	This paper	PDB: 8HLW

**Experimental models: Cell lines**		

HEK293T	ATCC	ACS-4500
U251 MG	Merk	09063001

**Oligonucleotides**		

SIRT1 siRNA	Santa Cruz	sc-40986
SIRT2 siRNA	Santa Cruz	sc-40988
SIRT3 siRNA	Santa Cruz	sc-61555
HDAC3 siRNA	Santa Cruz	sc-35538
Control siRNA	Santa Cruz	sc-37007

**Software and algorithms**		

MicroCal PEAQ-ITC analysis v141	Malvern	https://www.malvernpanalytical.com.cn/support/product-support/software/microcal-peaq-itc-analysis-software-v141
Image lab	Bio-Rad Laboratories, lnc.	https://www.bio-rad.com/en-hk/category/image-lab-software-resources?ID=PJWA0VTU86LJ
MaxQuant v1.6.14.0	N/A	https://www.maxquant.org
HKL2000	Otwinowski et al.^31^	http://www.hkl-xray.com/
Phaser	McCoy et al.^32^	https://www.ccp4.ac.uk/html/phaser.html
Coot v7.0	Emsley et al.^33^	https://www2.mrc-lmb.cam.ac.uk/personal/pemsley/coot/
Prism v9.4.0	GraphPad Software, Inc.	https://www.graphpad.com/features
LIGPLOT v2.2.5	Laskowski et al.^34^	https://www.ebi.ac.uk/thornton-srv/software/LigPlus/

### Resource availability

#### Lead contact

Further information and requests for resources and reagents should be directed to and will be fulfilled by the lead contact, Zhuming Fan (fanzm@ihep.ac.cn).

#### Materials availability

This study did not generate new unique reagents.

### Experimental model and study participant details

#### Cell lines

HEK293T (*Homo sapiens*) and U251 MG (*Homo sapiens*) cell lines were cultured in DMEM (Dulbecco’s Modified Eagle Medium) (high glucose), and supplemented with 10% fetal bovine serum (FBS) at 37°C under 5% CO2 condition.

Only the lysate of U251 MG cell was utilized for the pull-down assay of p-H4K16laAlk (no physiological function experiments). While HEK293T cells were tested for mycoplasma negativity through the services provided by the Center for PanorOmic Sciences (CPOS) at the University of Hong Kong.

#### Bacterial strains for protein expression

BL21-CodonPlus (DE3)-RIL competent cells were cultured in 2×YT medium (5 g sodium chloride, 10 g yeast extract, and 16 g tryptone per liter) at 37°C (for amplification) or 16°C (for protein expression) with shaking at 220 rpm.

### Method details

#### Preparation of recombinant human Sirtuins

To prepare for the enzymatic assays and crystallization, the human SIRT1 core region (234–505 aa), SIRT2 (50–356 aa), SIRT5 (32–302), and full-length SIRT7 were cloned into a pET-28a-sumo vector, while SIRT3 (118–399 aa) and full-length SIRT6 were cloned into a pET-N-6xHis-TEV (Tobacco etch virus) vector. These vectors were then transformed and expressed in BL21-CodonPlus (DE3)-RIL competent cells. The recombinant proteins were purified via affinity and ion exchange purification, followed by gel filtration. To remove tags during purification, TEV, and sumo protease were utilized. The purified recombinant human Sirtuins were then concentrated to a final concentration of 10 mg/mL and stored at −80°C freezer for use in the following experiments.

#### NAD^+^ consumption/cycling assay

To screen the delactylation activity of Sirtuins, we employed a modified^35^ NAD^+^ cycling assay to measure the amount of unused NAD^+^ levels after Sirtuins’ consumption reactions. This assay involves the conversion of NAD^+^ to NADH by alcohol dehydrogenase, followed by the conversion of NADH to NAD^+^ by NADH oxidoreductase, diaphorase with resazurin as an electron acceptor. The reduced resazurin is then measured as resorufin, which has a high fluorescence characteristic.^36^

To perform the NAD^+^ consumption assay, 50 μM recombinant Sirtuins were co-incubated with 500 μM Kla peptides and 100 μM NAD^+^ in 150 NaCl, 20 mM Tris (pH = 7.5) buffer for 3 h or overnight at 37°C. Then, hydrochloric acid, final concentration at 150 mM, was added to terminate the reaction. As for the cycling assay, 200 μL reaction buffer containing 100 mM Na_2_HPO_4_ (pH = 8.0), 10 μM resazurin, 10 μM flavin mononucleotide (FMN), 1% absolute ethanol, 66.5 μg/mL alcohol dehydrogenase and 5 μg/mL diaphorase was simultaneously mixed with 10 μL of NAD^+^ consumption mixtures in 96-well white plate. Then, the plate was immediately measured by fluorescence spectrophotometer under excitation 549 nm, emission 576 nm condition. Each histogram displayed represents one independent NAD^+^ consumption/cycling assay. (Lactyl peptides used in NAD^+^ consumption assay are listed in Table S5).

#### Binding affinity measurement

ITC was performed to determine the binding affinity between Sirtuins and Kla peptides. Briefly, 200 μL of 30 μM of recombinant Sirtuins were loaded into the “cell” of the ITC, and 40 μL of 2 mM lactyl peptides were loaded into the “needle” of the ITC. The proteins and freeze-dried peptides were then equilibrated into the same buffer condition, 20 μM of Tris (pH = 7.5), and 150 mM NaCl via dialysis and dissolution. After 10 min equilibration at 25°C, 40 μL of peptides divided into 20 injections were titrated into the “cell.” The ITC data and binding affinity were analyzed and calculated using the MicroCal PEAQ-ITC analysis software. (Lactyl peptides used in ITC assay are listed in Table S6).

#### LC-MS analysis for Sirtuins and lactyl peptides

LC-MS analysis was performed to test and verify the delactylation activity of Sirtuins. The lactyl peptides used in LC-MS assay was added 2 additional tryptophan residues at the N- and C- terminus to enhance the HPLC absorption sensitiveness. After incubating the peptides with Sirtuins/HDAC3 at 37°C for 3 h. The reaction was halted by methanol, and LC-MS was used to analyze the erased ratio of different peptides. HPLC condition: peptides: 100 μM; HDAC3/NCOR2 and Sirtuins: 200 nM; 30–100% acetonitrile (ACN) 3–30 min for H4K16la peptide; 3–100% ACN 3–20 min for other lactyl peptides; column used: C18-Pro, 4.6 × 250 mm, 5 μm, 300 Å, J&K Scientific. (Lactyl peptides used in LC-MS analysis are listed in Table S7).

#### Kinetic study for SIRT3-H4K16la

To perform the kinetic study, the erasing rate was determined using the HPLC method. The *K*_*M*_ and *k*_*cat*_ values were then computed using the Lineweaver-Burk method.^37^ In brief, the H4K16la peptide (10, 20, 40, 60, 80, 100, 150, 200 μM) was incubated with SIRT3 (200 nM) in Tris (pH = 7.4) buffer at 37°C for 40 min. The erased rate was evaluated by HPLC, and integrations at 280 nm were recorded to give the erased ratio. The kinetic parameters were then obtained using the formula: $\frac{1}{v}=\left(\frac{K_{M}}{V_{\max}}\right)\left(\frac{1}{\left[S\right]}\right)+\frac{1}{V_{\max}}$.

#### siRNA knocking down assay

To investigate whether Sirtuins can erase histone Kla intracellularly, we conducted an siRNA knocking down assay on HEK 293T cells. Commercial siRNAs were purchased and transfected into HEK293T cells. For transfection, 0.8 million cells per well were seeded into a 6-well plate. 12 h later, after cells had adhered to the bottom of the well, the siRNA mixture (consisted of 3 μL siRNA pre-mixed with 9 μL Lipofectamine RNAiMAX in 150 μL of Opti-MEM medium) was added to the cell supernatant. After 48 h of incubation, cells were washed with PBS and lysed with RIPA (radio-immunoprecipitation assay) lysis reagent and sonication. The expression levels of Sirtuins/HDAC3 and H4K16la were measured via Western Blot.

#### Proteomic analysis using p-H4K16laAlk

##### p-H4K16laAlk probe synthesis

The probe p-H4K16laAlk was synthesized according to the method shown in Figure S17. In brief, peptide GGAK(mtt)R(pbf)H(trt)R(pbf)K(Boc)-NH_2_ was first synthesized using the solid phase method. Then 4.0 eq of 3-(3-(But-3-yn-1-yl)-3H-diazirin-3-yl)propanoic acid was added with 4.0 eq HATU, 4.0 eq HOAT and 4.0 eq DIEA. After overnight incubation, the resins were washed with DCM, MeOH and DMF three times. Then 1% TFA in DCM was added to remove the mtt group. After washing, 5.0 eq of lactic acid, 5.0 eq HATU, 5.0 eq HOAT and 10.0 eq DIEA was added to the resins. The final probe was obtained after cleavage and purified using HPLC. (Figure S18).

##### Preparation of LC-MS/MS samples

U251 cell lysate was incubated with 10 μM p-H4K16laAlk, 10 μM p-H4K16laAlk mixed with 100 μM H4K16la peptide (as the competitor group), and DMSO (as the negative control). These mixtures were then incubated at 37°C for 1 h. The mixtures were subjected to photo-cross-linking by irradiation with 365 nm UV for 5 min on ice. Subsequently, CuSO_4_, THPTA (tris-hydroxypropyltriazolylmethylamine), TCEP (tris(2-carboxyethyl)phosphine), and biotin azide were added, and the mixture was stirred vigorously for 2 h to click the alkynyl group to the biotin. After that, chilled acetone was added to remove non-protein components. The resulting precipitate was washed with chilled methanol, then redissolved with 1% SDS in PBS. The mixture was centrifuged again at 14000 rpm for 15 min to remove the insoluble precipitate. Next, 100 μL of streptavidin agarose beads (Thermo Fisher, 20353) were added to each tube, and incubated overnight at 4°C. The supernatant was discarded after centrifugation, and the beads were washed with 0.5% SDS in PBS, 0.1% SDS in PBS, and PBS three times (shaking for 15 min each time). The supernatant was removed using 1000 rpm centrifugation. Finally, 50 μL of loading buffer was added to each tube to dissolve the proteins bound to beads. And after 14000 rpm centrifugation, the supernatant was collected and boiled for SDS-page running.

##### In-gel trypsin digestion of LC-MS/MS samples

To analyze the protein samples, we resolved them by SDS-page gel and visualized them using Coomassie stain. Both gel lanes were then cut into slices and subjected to in-gel digestion. The gel slices were first reduced and alkylated by adding 10 mM TCEP and 55 mM 2-chloroacetamide (CAA), respectively. Protein digestion was then performed by incubating the slices with 1 ng/μL trypsin overnight at 37°C. The tryptic peptides were then extracted from the gel with 50% ACN/5% formic acid (FA) and 100% ACN sequentially. The peptide extracts were pooled together and dried using the SpeedVac. The peptides were subsequently desalted using C18 StageTips in preparation for LC-MS/MS analysis.

##### Data acquisition of LC-MS/MS samples

After eluting the peptides, we analyzed them using nano elute UHPLC, which was coupled to a Bruker times TOF pro mass spectrometer. The peptide mixture was loaded onto an Aurora C18 UHPLC column, with dimensions of 75 μm in diameter and 25 cm in length, and a particle size of 1.6 μm (IonOpticks, Australia). Chromatographic separation was achieved using a linear gradient of 2–30% of buffer B (0.1% FA in ACN) at a flow rate of 300 nL/min over 27 min. MS data was collected over a mass-to-charge (m/z) range of 100–1700, and MS/MS data was collected over the same range. During MS/MS data collection, each TIMS cycle lasted 1.1 s and included 1 MS scan plus an average of 10 PASEF MS/MS scans.

#### Synthesis and Evaluation of the fluorogenic probe, p-H4K16laNBD

To synthesize the fluorogenic probe, we first synthesized the peptide sequence GGAK(lac)RHRPra-NH_2_ using a solid phase method. After purification, the peptide was clicked with 5 mg of NBD-azide. We then added 1.0 equivalent CuSO_4_ to 2.0 equivalents THPTA and reduced it by 4.0 equivalents of ascorbic acid to form Cu(I) as a catalyst. After reacting for 4 h, the reaction mixture was purified by HPLC to give the final product, p-H4K16laNBD. The probe was verified by HPLC-Mass analysis, as shown in Figure S19.

To determine the absorption spectra, 10 μM p-H4K16laNBD was incubated with or without 0.2 μM SIRT3 and 100 μM NAD^+^ for 2 h in 20 mM HEPES buffer (pH = 8.0). For the fluorescence assay, the fluorescence was detected using an excitation wavelength of 480 nm, and the emitted fluorescence signal was measured over the range of 520–740 nm using a microplate reader (Molecular Devices SpectraMax ID5 Microplate Reader).

For the HPLC analysis, we incubated 10 μM p-H4K16laNBD with 0.2 μM SIRT3 and 100 μM NAD^+^ in 20 mM HEPES buffer (pH = 8.0) for 2 h. We also performed time-dependent fluorescence measurements by incubating 10 μM p-H4K16laNBD with 0.2 μM SIRT3 and 100 μM NAD^+^ or 0.1 μM HDAC3 in 20 mM HEPES buffer (pH = 8.0) for different time intervals. The fluorescence was measured using an excitation wavelength of 480 nm, and the emitted fluorescence signal at 550 nm was measured using a microplate reader (Molecular Devices SpectraMax ID5 Microplate Reader).

To determine the fluorescence change after incubating p-H4K16laNBD with various enzymes (HDAC3, SIRT1-3, SIRT5-7), we incubated 10 μM p-H4K16laNBD with 0.2 μM SIRT1-7 (except SIRT4) in 20 mM HEPES buffer (pH = 8.0) containing 100 μM NAD^+^ for 2 h. For positive control, we used 0.1 μM HDAC3. The fluorescence was measured using an excitation wavelength of 480 nm, and the emitted fluorescence signal at 550 nm was measured using a microplate reader (Molecular Devices SpectraMax ID5 Microplate Reader).

#### Structure determination of the Sirtuin-Kla complex

To screen protein-peptide interactions, SIRT3 was mixed with lactyl peptides at a protein:peptide molar ratio of 1:5 and incubated for 2 h at room temperature. The protein-peptide mixtures were then subjected to sitting-drop assay, and crystals were formed after 2 days of incubation at 18°C.

Two crystal conditions, SIRT3-H3K23la(L) and SIRT3-H4K16la(L), were obtained, with the former in 0.1 M Bis-Tris pH 6.5 and 28% (w/v) Polyethylene glycol monomethyl ether 2000 condition, and the latter in 0.1 M Tris pH 8.5 and 25% (w/v) Polyethylene glycol 3350 condition. The crystals were optimized by adding glycerol and potassium iodide as additives for SIRT3-H3K23la(L) and SIRT3-H4K16la(L), respectively. Optimized crystals were briefly soaked in the original crystallization condition containing the cryoprotectant reagent before being flash-frozen by liquid nitrogen and stored in a liquid nitrogen tank.

The crystals were taken to the Shanghai Synchrotron Radiation Facilities BL19U beamline for X-ray diffraction. The diffraction images were initially processed using HKL 2000 software for indexing, integrating, and scaling. A high homology model from the PDB: 3GLS was used in molecular replacement, which was supported by “Phaser” in CCP4 suites to solve the phase problem. The solved structure and electron density map were refined using “Refmac” of CCP4 suites or Phenix and manually refined using Coot.

### Quantification and statistical analysis

To quantify the band intensity in the Western blot analysis, we utilized the Image lab software. The data are presented as mean ± SD (n ≥ 3). Statistical differences were evaluated using the two-tailed Student’s *t*-test. A significance level of ^∗^*p* < 0.05 and ^∗∗^*p* < 0.01 was considered statistically significant.

For proteomic analysis (LC-MS/MS samples), we used MaxQuant 1.6.14.0 software to process the raw mass spectrometry data. The raw data was searched against the Human Swissprot FASTA database from May 2022, which contained 20,361 entries. The following settings were used: oxidized methionine (M) and acetylation (protein N-term) were selected as dynamic modifications, and carbamidomethyl© was used as the fixed modification. A minimum peptide length of 7 amino acids was enabled. Confident proteins were identified using a target-decoy approach with a reversed database, strict false-discovery rate (FDR) of 1% at peptide and peptide-spectrum matches (PSMs) levels, and a minimum of ≥1 unique peptide and ≥2 PSMs. The results were summarized and plotted (indicated as the ratio of Group_p-H4K16laAlk_ to Group_p-H4K16laAlk with 100 μM competitor_) using Prism (version 9.4.0), and submitted to iproX (PXD039731) database.

## Data Availability

• The raw mass spectrometry data has been deposited to the ProteomeXchange Consortium via the iProX partner repository with the dataset identifier PXD039731. And the crystal structures for SIRT3-H3K23la and SIRT3-H4K16la have been deposited to the Protein DataBank (PDB) with the respective codes 8HLY and 8HLW. • This paper does not report original code. • Additional information required for the reanalysis of the data reported in this paper is available from the lead contact upon request.
