# Supplementary material for: Identification of SIRT3 as an eraser of H4K16la
Source: iScience. 2023 Aug 28;26(10):107757. doi: 10.1016/j.isci.2023.107757 (PMC10504495; doi:10.1016/j.isci.2023.107757)
Supplement: Document S1. Figures S1–S19 and Tables S1–S7 [file mmc1.pdf]

iScience, Volume *26*

Supplemental information

Identification of SIRT3 as an eraser of H4K16la

Zhuming Fan, Zhiyang Liu, Nan Zhang, Wenyu Wei, Ke Cheng, Hongyan Sun, and Quan Hao

Contents

Figure S1. Final purification results of recombinant human Sirtuins, related to Figure 1
Figure S2. NAD$^+$ consumption/cycling assay, related to Figure 1A
Figure S3. ITC results of SIRT1 with different Kla peptides, related to Figure 1C
Figure S4. ITC results of SIRT2 with different Kla peptides, related to Figure 1C
Figure S5. ITC results of SIRT3 with different Kla peptides, related to Figure 1C
Figure S6. The mass spectrum results for HPLC analysis after incubating Sirtuins and HDAC3 (200 nM) with 100 μM H4K16la, related to Figure 1D, E
Figure S7. LC-MS analysis for SIRT1-3 and lactyl peptides, related to Figure 1
Figure S8. 3 replicates of HPLC analysis results for erasing rate determination, related to Figure 1F
Figure S9. ITC row data of SIRT3 titrated by L(A)/D(B)-H4K16la, related to Figure 1H
Figure S10. Western blot analysis of cellular H4K16la level change, related to Figure 1I
Figure S11. Western blot analysis of cellular H4K16la level change, related to Figure 1J
Figure S12. The amido linkage binding details of acyl-lysine with SIRT3, related to Figure 2
Figure S13. Detailed interactions in SIRT3-H3K23la structure, related to Figure 2
Figure S14. Chemical structure of the lactate probe (alkynyl-labelled mimics), related to Figure 3
Figure S15. 4 individual experiments of Western blot analysis of detecting the existence of SIRT3 in the pull-down products, related to Figure 3C
Figure S16. Flurogenic parameter of the p-H4k16laNBD probe, related to Figure 3
Figure S17. Synthesis procedure of the p-H4K16laAlk (upper) and the competitor (H4K16la peptide) (lower), related to STAR Methods
Figure S18. LC-MS analysis of the p-H4K16laAlk (upper) and the competitor (H4K16la peptide) (lower) , related to STAR Methods
Figure S19. Synthesis procedure and the LC-MS analysis of p-H4K16laNBD, related to STAR Methods

Table S1. The literature evidence of Sirtuins' preferred sites, related to Figure 1A
Table S2. The literature evidence of the nuclear existence of SIRT3, related to Figure 1I, J
Table S3. Crystal data collection and refinement statistics, related to Figure 2
Table S4. Proteomic analysis: protein identified after enrichment (with enriched ratio≥2, $p < 0.05$), related to Figure 3B
Table S5. Peptide sequences used in NAD$^+$ consumption/cycling assay, related to STAR Methods
Table S6. Peptide sequences used in ITC assay, related to STAR Methods
Table S7. Peptide sequences used in HPLC-MS analysis, related to STAR Methods

Reference

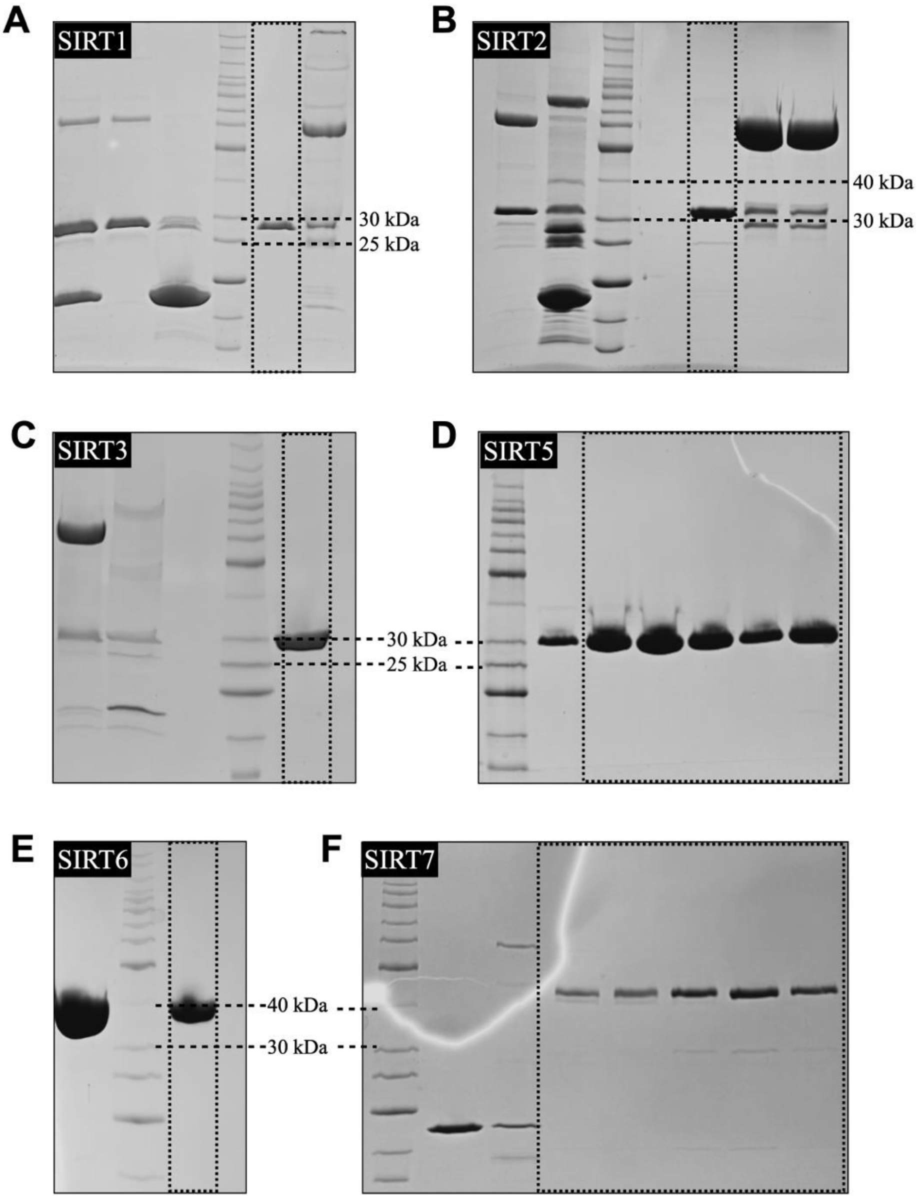

Figure S1. Final purification results of recombinant human Sirtuins, related to Figure 1

hSIRT1 core region (234-505 aa, 30.1 kDa). (B) SIRT2 (50-356 aa, 34.6 kDa). (C) SIRT3 (118-399 aa, 31.2 kDa). (D) SIRT5 (32-302, 29.5 kDa). (E) Full-length SIRT6 (39.1 kDa). (F) Full-length SIRT7 (44.9 kDa). Dot-framed pure fractions were collected, buffer exchanged and concentrated for the following enzymatic and crystallographic experiments.

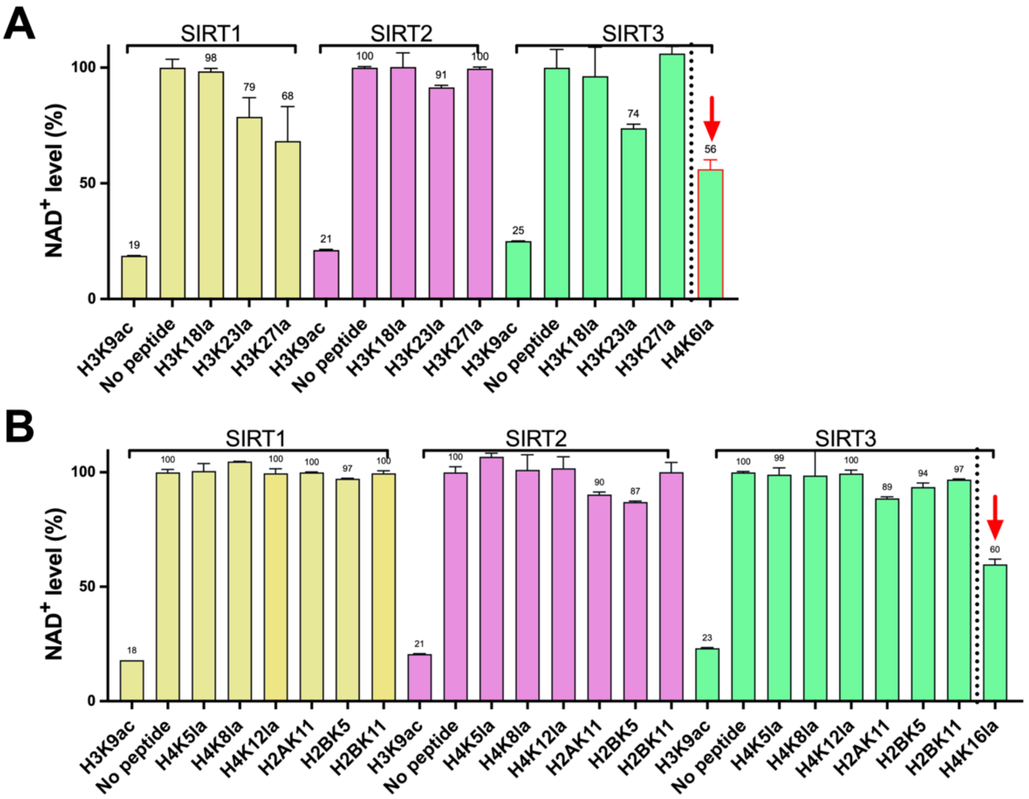

Figure S2. NAD⁺ consumption/cycling assay, related to Figure 1A

(A) 3 h NAD⁺ consumption/cycling assay of SIRT1-3 on H3K18la, H3K23la, H3K27la and H4K16la (SIRT3 only); (B) 3 h NAD⁺ consumption/cycling assay of SIRT1-3 on H4K5la, H4K8la, H4K12la, H2AK11la, H2BK5la, H2BK11la, and H4K16la (SIRT3 only).

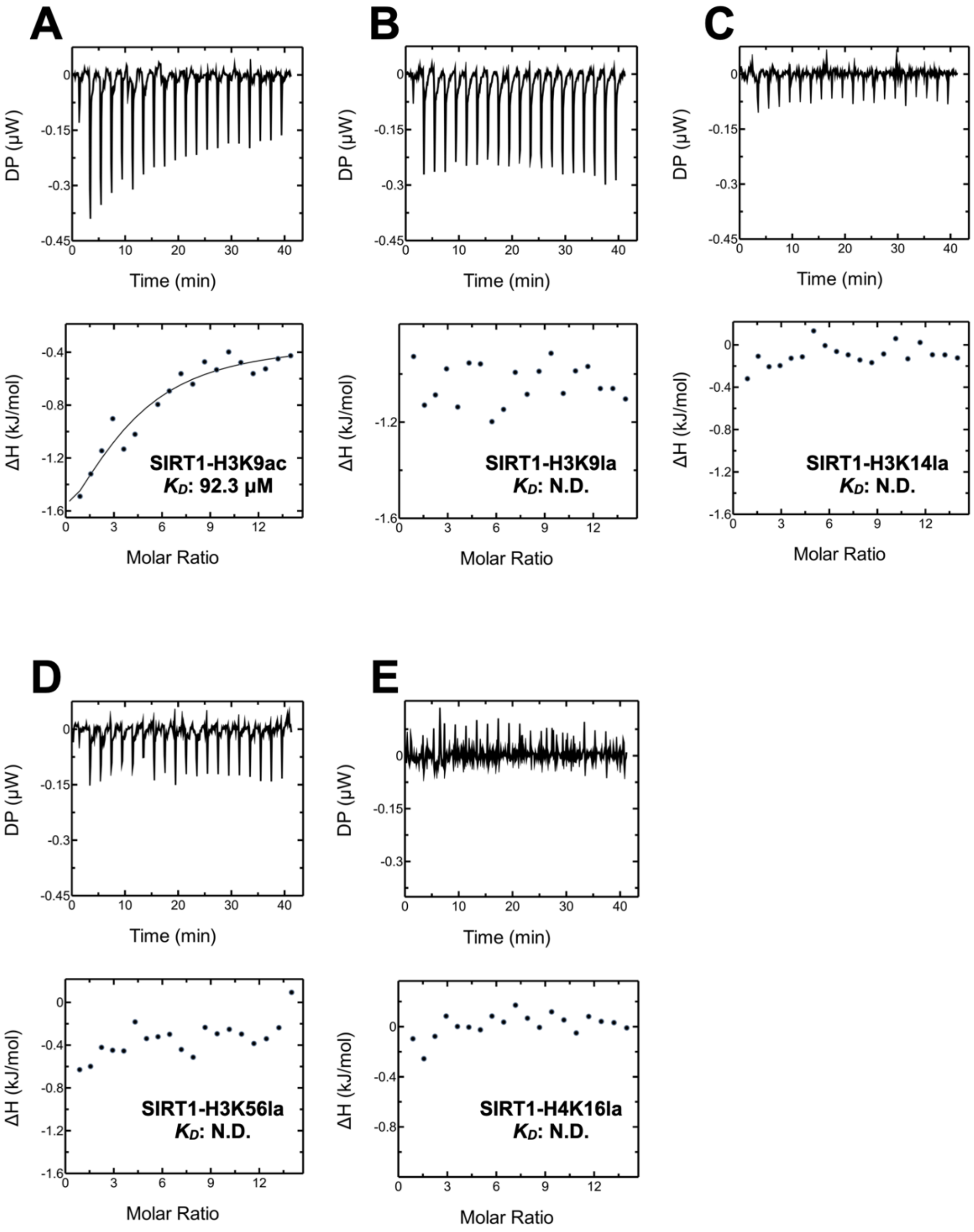

Figure S3. ITC results of SIRT1 with different Kla peptides, related to Figure 1C

40 µL, 2 mM of H3K9ac as the positive control (A), H3K9la (B), H3K14la (C), H3K56la (D) and H4K16la (E) were divided into 20 injections titrated into 200 µL, 30 µM of SIRT1. ITC data and binding affinity were analyzed by MicroCal PEAQ-ITC analysis software.

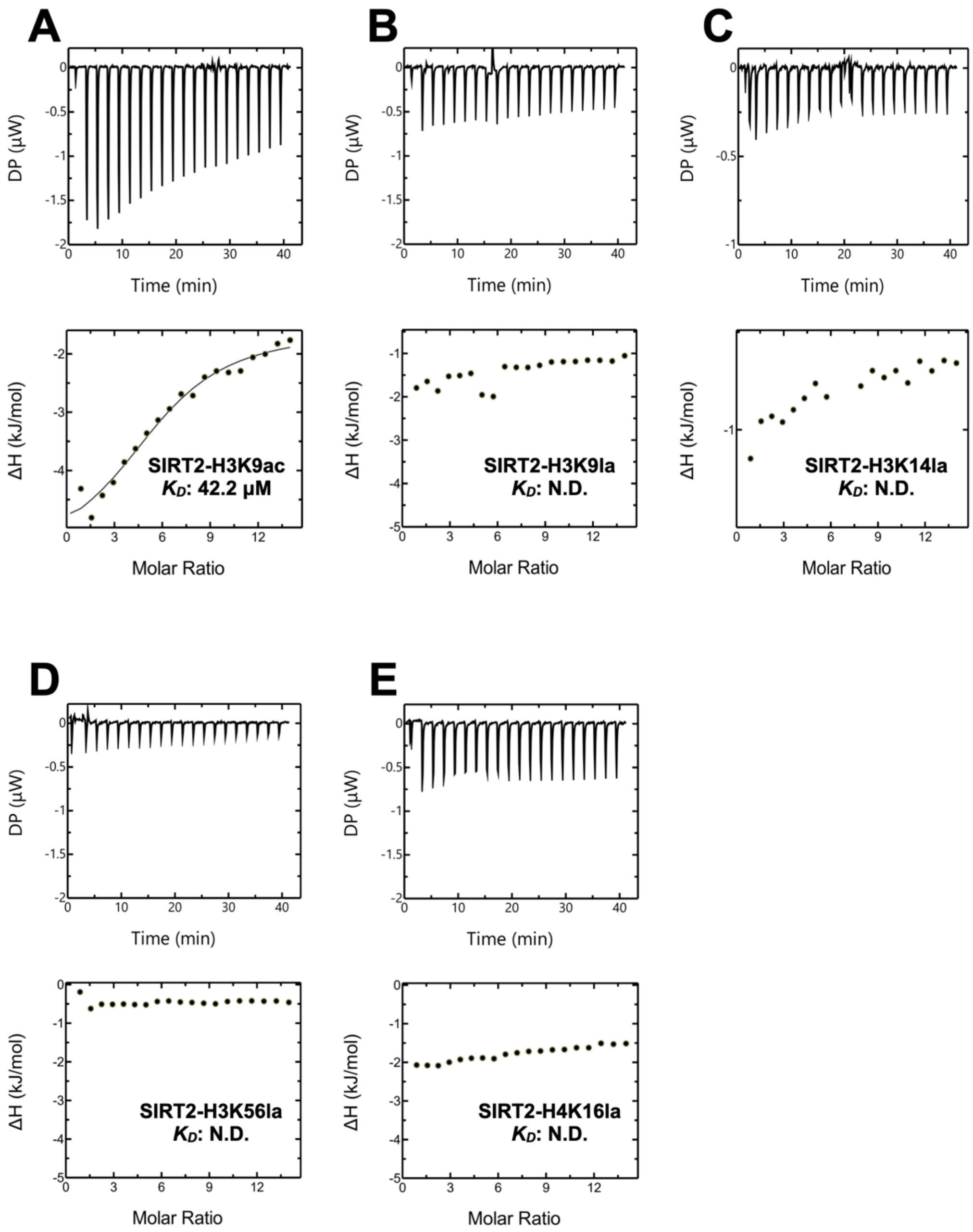

Figure S4. ITC results of SIRT2 with different Kla peptides, related to Figure 1C

40 μL, 2 mM of H3K9ac as the positive control (A), H3K9la (B), H3K14la (C), H3K56la (D) and H4K16la (E) were divided into 20 injections titrated into 200 μL, 30 μM of SIRT2. ITC data and binding affinity were analyzed by MicroCal PEAQ-ITC analysis software.

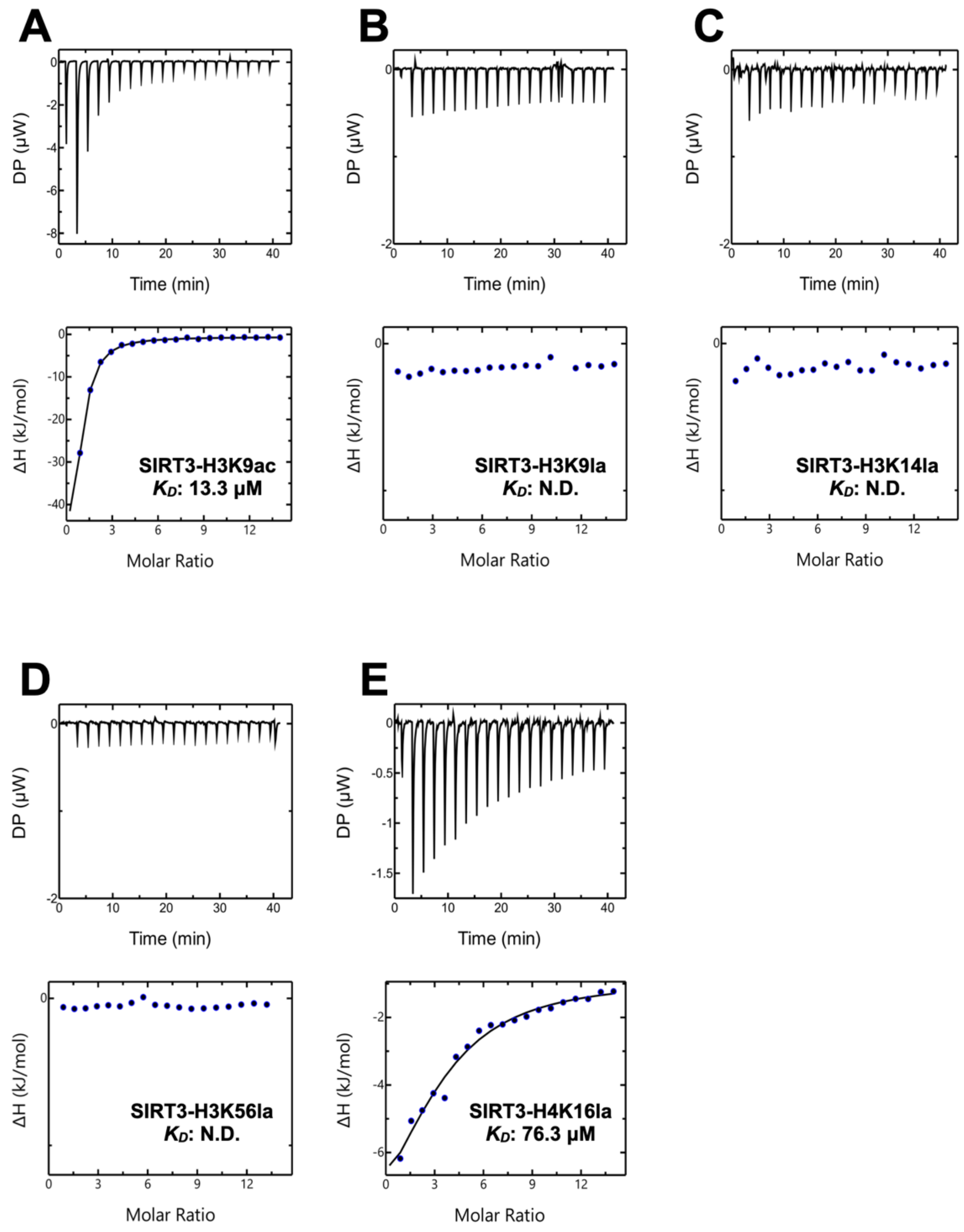

Figure S5. **ITC results of SIRT3 with different Kla peptides, related to Figure 1C**
40 μL, 2 mM of H3K9ac as the positive control (A), H3K9la (B), H3K14la (C), H3K56la (D) and H4K16la (E) were divided into 20 injections titrated into 200 μL, 30 μM of SIRT3. ITC data and binding affinity were analyzed by MicroCal PEAQ-ITC analysis software.

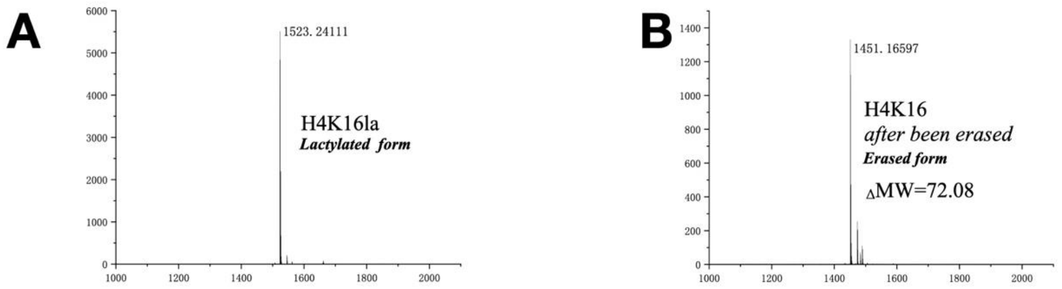

Figure S6. The mass spectrum results for HPLC analysis after incubating Sirtuins and HDAC3 (200 nM) with 100 μM H4K16la, related to Figure 1D, E

(A)H4K16la, (B)H4K16 (modification after being erased). HPLC condition: peptide, 100 μM; protein, 200 nM; 30-100% ACN 3-30 min. The column used: C18-Pro, 4.6x250 mm, 5 μm, 300 Å, J&K Scientific.

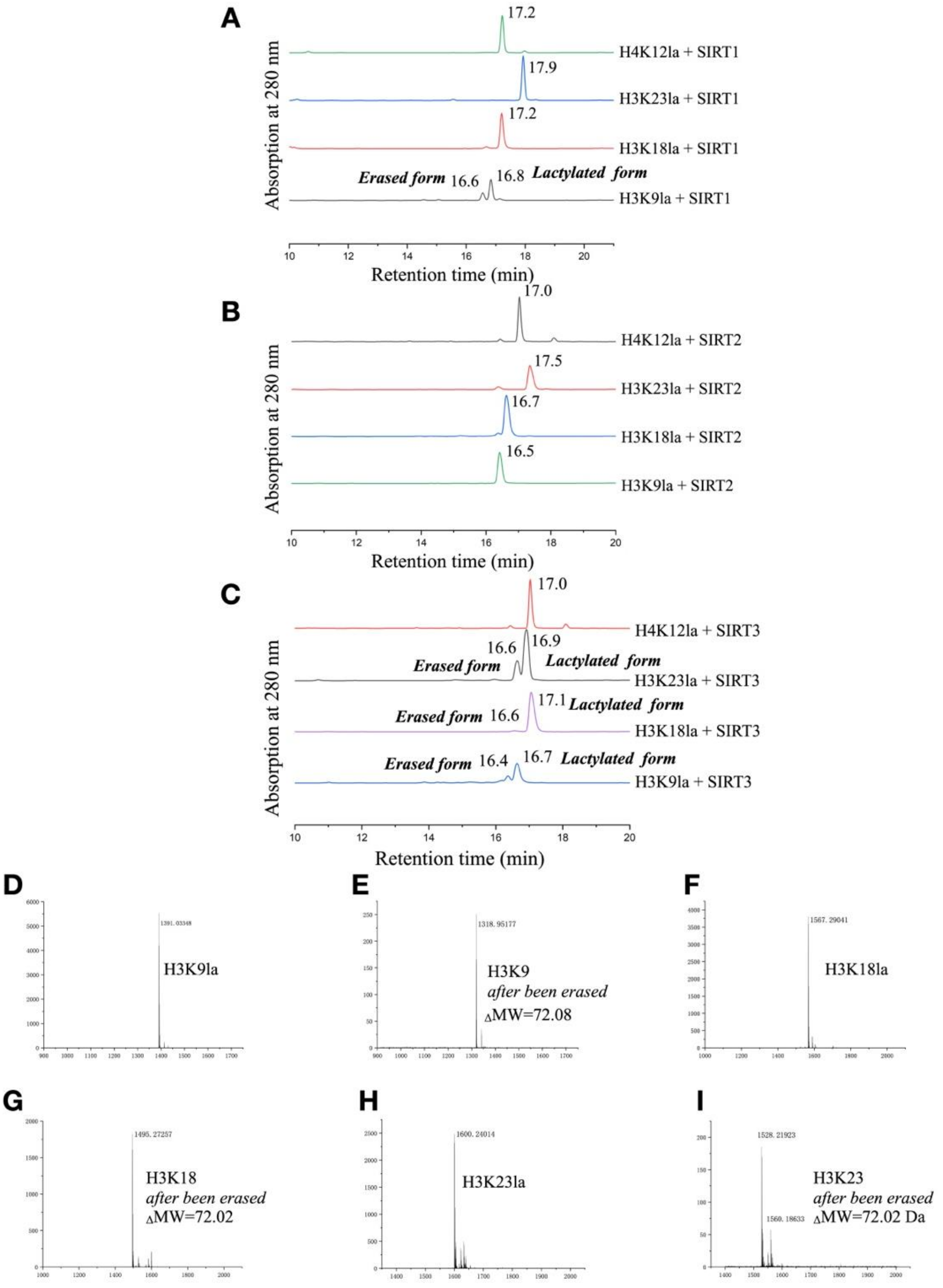

Figure S7. LC-MS analysis for SIRT1-3 and lactyl peptides, related to Figure 1

(A) Comparison of the SIRT1 erasing capacity against H3K9la, H3K18, H3K23la, and H4K12la. (3 hours incubation) (B) Comparison of the SIRT2 erasing capacity against H3K9la, H3K18, H3K23la, and H4K12la. (3 hours incubation) (C) Comparison of the SIRT3 erasing capacity against H3K9la, H3K18, H3K23la, and H4K12la. (3 hours incubation) (D-I) The mass result of H3K9la, H3K18, and H3K23la and their modification-erased form. HPLC condition: peptide, 100 μM; protein, 200 nM; 3-100% ACN 3-20 min. The column used: C18-Pro, 4.6x250 mm, 5 μm, 300 Å, J&K Scientific.

Replicate-1

H4k16 10μM

Name	Retention Time	Area	% Area	Height
1	10.578	51858	25.84	4987
2	10.996	148823	74.16	13550

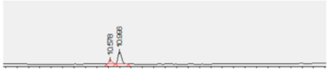

Calculated v=0.065μmol/L/min

H4k16 20μM

Name	Retention Time	Area	% Area	Height
2	10.965	315503	76.43	32780
1	10.577	97316	23.57	8599

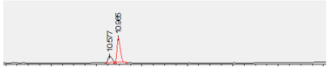

Calculated v=0.118μmol/L/min

H4k16 40μM

Name	Retention Time	Area	% Area	Height
1	10.550	102263	20.92	8845
2	10.915	386655	79.08	36333

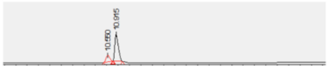

Calculated v=0.209μmol/L/min

H4k16 60μM

Name	Retention Time	Area	% Area	Height
2	10.904	570454	83.33	56829
1	10.490	114136	16.67	9412

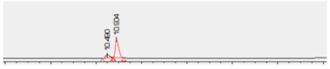

Calculated v=0.250μmol/L/min

H4k16 80μM

Name	Retention Time	Area	% Area	Height
2	11.106	425090	84.82	36315
1	10.761	76054	15.18	7567

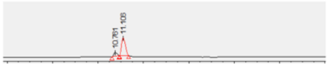

Calculated v=0.304μmol/L/min

H4k16 100μM

Name	Retention Time	Area	% Area	Height
1	10.565	191917	15.77	16178
2	10.935	1025237	84.23	81046

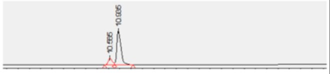

Calculated v=0.394μmol/L/min

H4k16 150μM

Name	Retention Time	Area	% Area	Height
2	10.893	2084217	86.06	199750
1	10.547	337609	13.94	31267

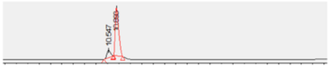

Calculated v=0.523μmol/L/min

H4k16 200μM

Name	Retention Time	Area	% Area	Height
2	10.659	1892504	87.16	138714
1	10.061	278850	12.84	18477

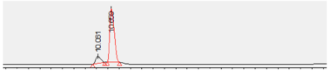

Calculated v=0.642μmol/L/min

Replicate-2

H4K16la 10μM

Name	Retention Time	Area	% Area	Height
1	10.586	18420	18.79	1650
2	11.003	79600	81.21	6738

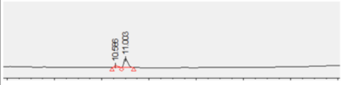

v=0.0469μmol/L/min

H4K16la 20μM

Name	Retention Time	Area	% Area	Height
1	10.570	25274	15.81	2112
2	10.892	134633	84.19	10629

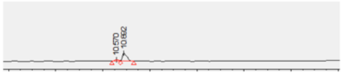

v=0.0791μmol/L/min

H4K16la 40μM

Name	Retention Time	Area	% Area	Height
1	10.466	120383	18.52	9806
2	10.870	529570	81.48	39439

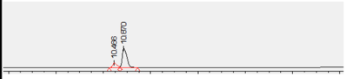

v=0.1852μmol/L/min

H4K16la 60μM

Name	Retention Time	Area	% Area	Height
1	10.353	340815	17.85	28088
2	10.799	1568537	82.15	126560

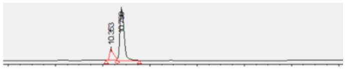

v=0.2678μmol/L/min

H4K16la 80μM

Name	Retention Time	Area	% Area	Height
1	10.443	283235	17.20	18877
2	10.831	1363443	82.80	100937

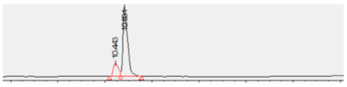

v=0.3440μmol/L/min

H4K16la 100μM

Name	Retention Time	Area	% Area	Height
1	10.604	261926	17.69	23220
2	10.967	1218438	82.31	91310

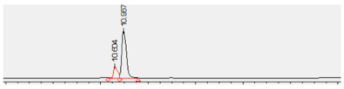

v=0.4423μmol/L/min

H4K16la 150μM

Name	Retention Time	Area	% Area	Height
2	10.841	2130245	81.98	177176
1	10.409	468153	18.02	32447

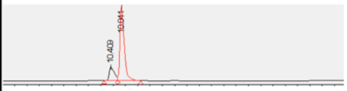

v=0.6758μmol/L/min

H4K16la 200μM

Name	Retention Time	Area	% Area	Height
2	10.858	2474663	83.35	204108
1	10.465	494357	16.65	40986

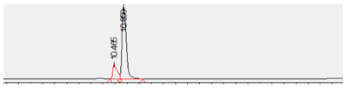

v=0.8325μmol/L/min

Replicate-3

H4K16la 10μM

Name	Retention Time	Area	% Area	Height
1	10.604	112486	26.92	9786
2	11.005	305371	73.08	31250

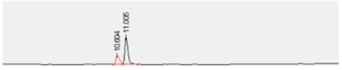

Calculated v=0.067μmol/L/min

H4K16la 20μM

Name	Retention Time	Area	% Area	Height
2	10.967	363878	76.35	38246
1	10.534	112743	23.65	10814

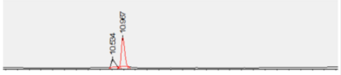

Calculated v=0.118μmol/L/min

H4K16la 40μM

Name	Retention Time	Area	% Area	Height
1	10.533	156513	18.38	11417
2	10.909	694963	81.62	60341

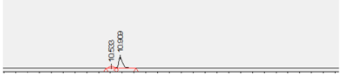

Calculated v=0.183μmol/L/min

H4K16la 60μM

Name	Retention Time	Area	% Area	Height
2	10.970	566994	83.23	50818
1	10.593	114245	16.77	12306

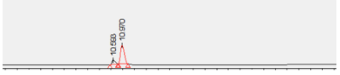

Calculated v=0.252μmol/L/min

H4K16la 80μM

Name	Retention Time	Area	% Area	Height
2	10.905	617948	82.87	59249
1	10.501	127696	17.13	10567

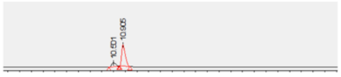

Calculated v=0.343μmol/L/min

H4K16la 100μM

Name	Retention Time	Area	% Area	Height
1	10.565	191917	15.77	16178
2	10.935	1025237	84.23	81046

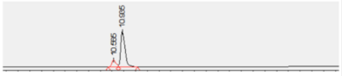

Calculated v=0.394μmol/L/min

H4K16la 150μM

Name	Retention Time	Area	% Area	Height
1	10.525	141240	13.20	15658
2	10.910	928473	86.80	80557

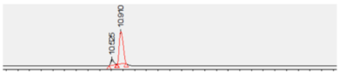

Calculated v=0.495μmol/L/min

H4K16la 200μM

Name	Retention Time	Area	% Area	Height
1	10.833	371781	14.52	33804
2	11.088	2189386	85.48	198834

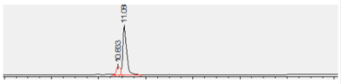

Calculated v=0.726μmol/L/min

Figure S8. 3 replicates of HPLC analysis results for erasing rate determination, related to Figure 1F

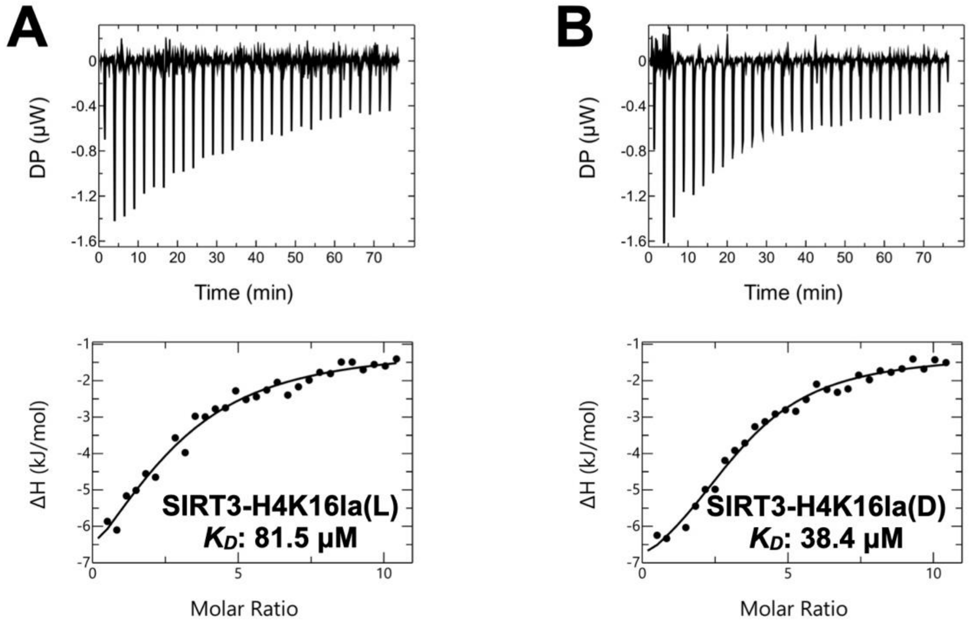

Figure S9. ITC row data of SIRT3 titrated by L(A)/D(B)-H4K16la, related to Figure 1H
ITC data and binding affinity were analyzed by MicroCal PEAQ-ITC analysis software.

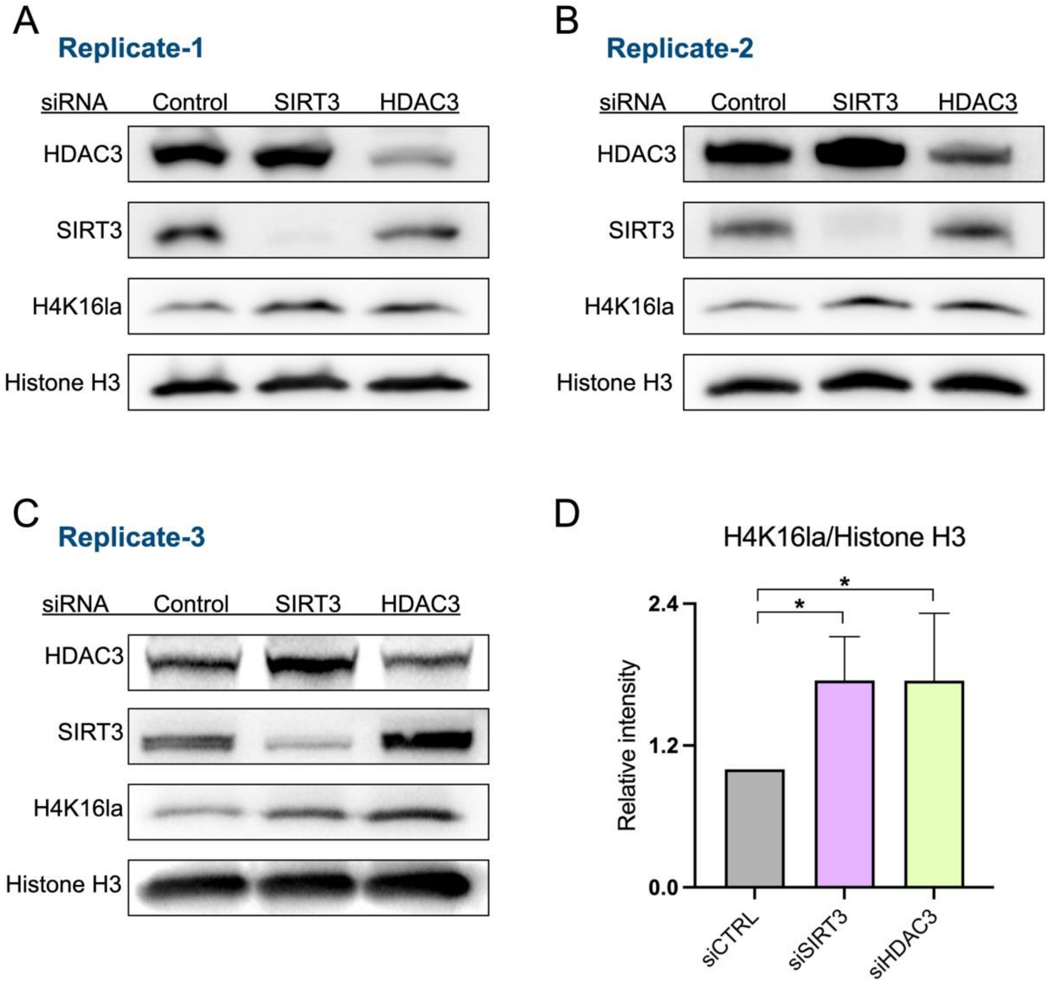

Figure S10. Western blot analysis of cellular H4K16la level change, related to Figure 1I

After 48 h post-knockdown of SIRT3 and HDAC3 via siRNA in HEK293T cells, increased H4K16la level was detected. (A-C) 3 western blot replicates of this experiment. (D) Histone H3 was used as the loading control. Values represent mean ± SD. Statistical comparisons between groups were analyzed using the Student *t* test. $^*p<0.05$.

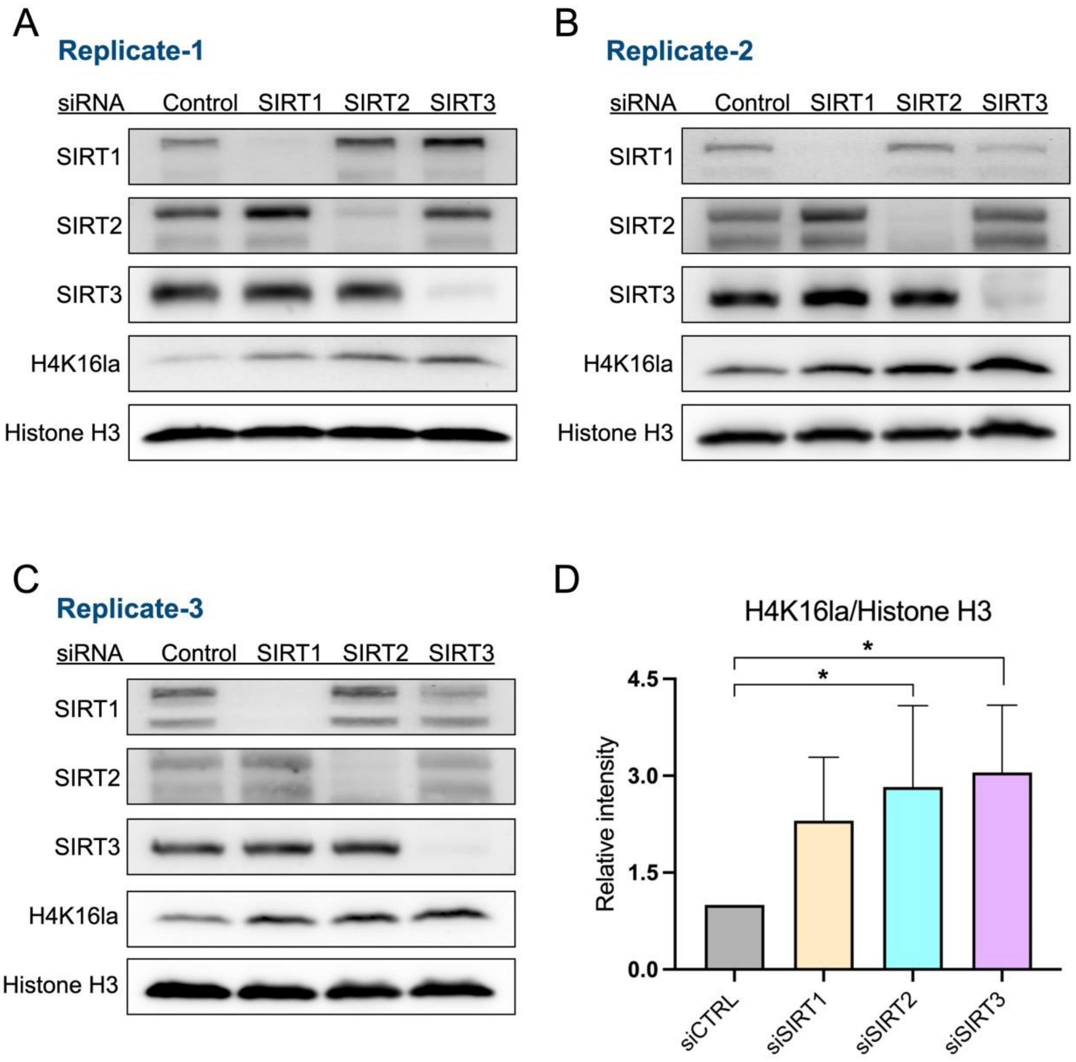

Figure S11. Western blot analysis of cellular H4K16la level change, related to Figure 1J

After 48 h post-knockdown of SIRT1-3 via siRNA in HEK293T cells, increased H4K16la level was detected. (A-C) 3 western blot replicates of this experiment. (D) Histone H3 was used as the loading control. Values represent mean ± SD. Statistical comparisons between groups were analyzed using the Student *t* test. *p<0.05.

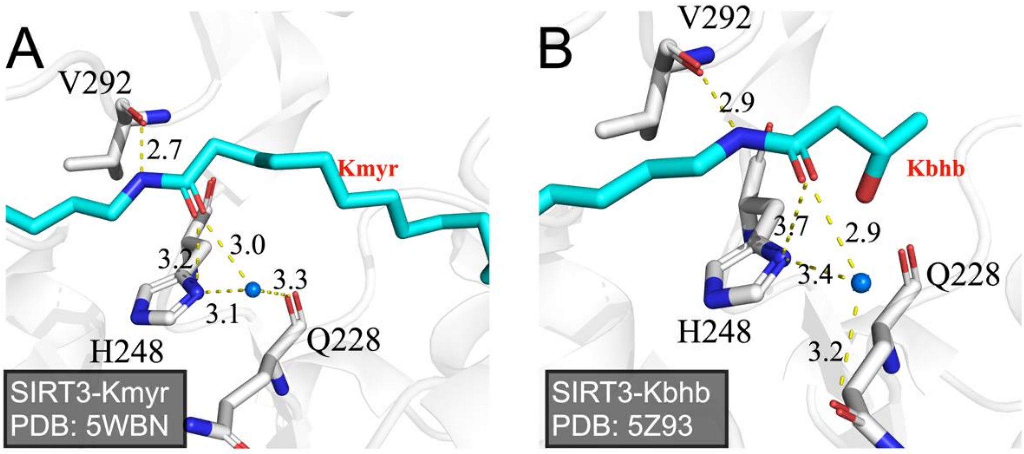

Figure S12. The amido linkage binding details of acyl-lysine with SIRT3, related to Figure 2

(A) PDB 5WBN, SIRT3 in complex with Kmyr peptide. (B) PDB 5Z93, SIRT3 in complex with Kbhb peptide. SIRT3 (grey), acyl-lysine peptides (cyan), water (marine sphere). Hydrogen bond is represented as a dashed line. The length (Å) of the hydrogen bond is labelled next to the dashed line.

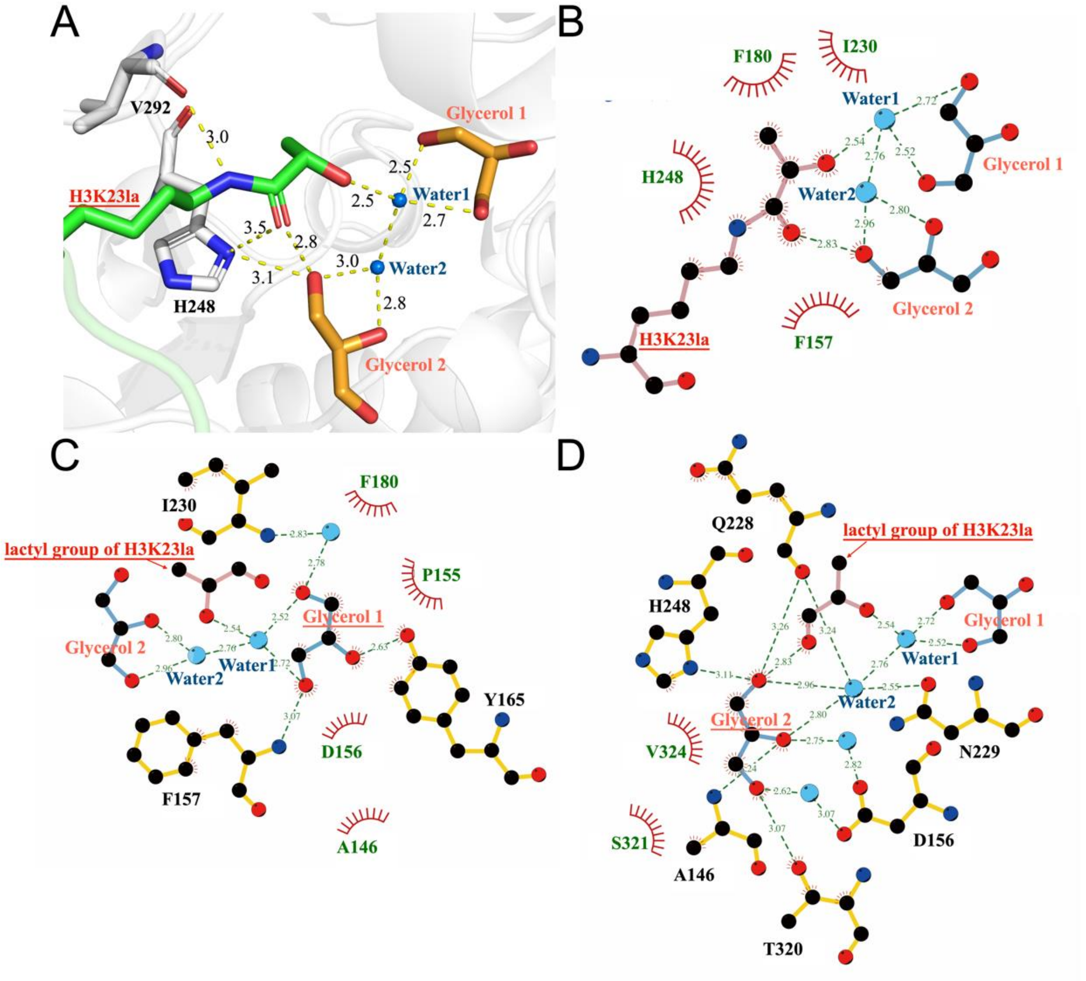

Figure S13. Detailed interactions in SIRT3-H3K23la structure, related to Figure 2

(A) Structure depicting details of the interaction between SIRT3 (white), H3K23la(L) (green), water molecules (marine spheres) and glycerol molecules (orange). (B-D) LIGPLOT diagram describing interactions of surrounding residues and molecules towards the (B) H3K23(L) ligand, (C) glycerol molecule 1 (Glycerol 1) and (D) glycerol molecule 2 (Glycerol 2). H3K23la(L) (bond colour: light pink), glycerol molecules (bond colour: blue) and SIRT3 (bond colour: gold) are depicted in ball-and-stick mode. Carbon is represented as a black ball; nitrogen is represented as a blue ball; oxygen is represented as a red ball; water molecule is represented as a marine sphere; hydrophobic interactions are shown as red arcs; hydrogen bond is represented as a dashed line. The length (Å) of the hydrogen bond is labelled next to the dashed line.

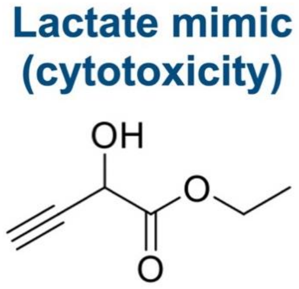

Figure S14. Chemical structure of the lactate probe (alkynyl-labelled mimics), related to Figure 3

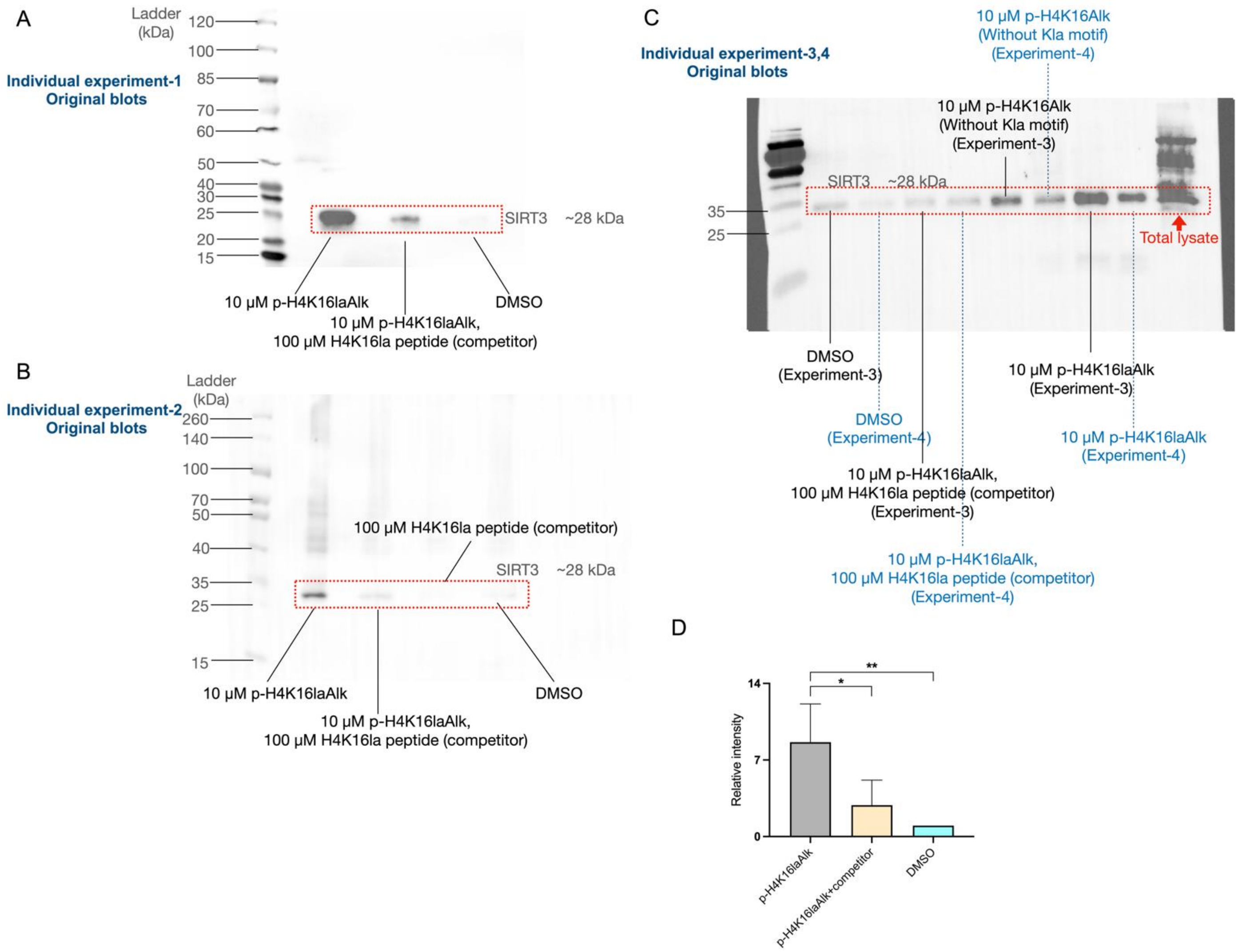

Figure S15. 4 individual experiments of Western blot analysis of detecting the existence of SIRT3 in the pull-down products, related to Figure 3C

(A) Experiment-1: western blot analysis of detecting the existence of SIRT3 in the pull-down products of group$_{\text{p-H4K16laAlk}}$, group$_{\text{p-H4K16laAlk with competitor}}$, and DMSO group (negative control). (B) Experiment-2: western blot analysis of detecting the existence of SIRT3 in the pull-down products of group$_{\text{p-H4K16laAlk}}$, group$_{\text{p-H4K16laAlk with competitor}}$, group$_{\text{competitor}}$, and DMSO group (negative control). (C) Experiment-3, 4: western blot analysis of detecting the existence of SIRT3 in the pull-down products of DMSO group (negative control), group$_{\text{p-H4K16laAlk with competitor}}$, group$_{\text{p-H4K16Alk}}$ (probe without lactylation modification), and group$_{\text{p-H4K16laAlk}}$. (D) Values represent mean ± SD. Statistical comparisons between groups were analyzed using the Student *t* test. **p*<0.05, ***p*<0.01.

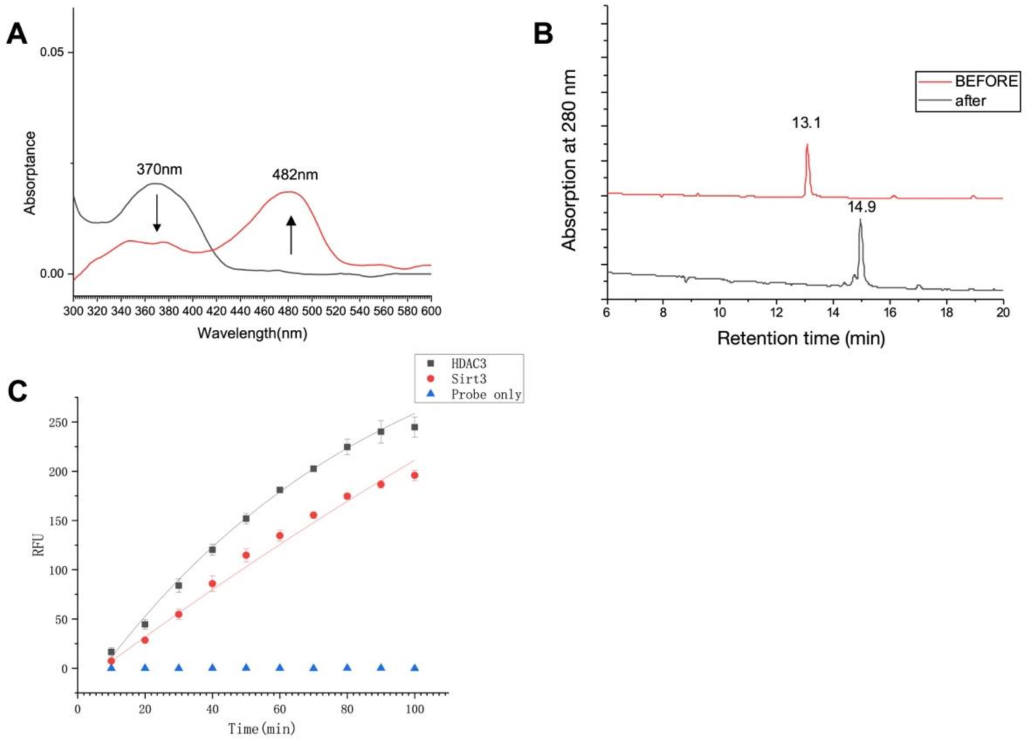

Figure S16. Flurogenic parameter of the p-H4k16laNBD probe, related to Figure 3
(A) Absorption spectra of p-H4K16laNBD probe (10 μM) before (grey) and after (red) enzymatic reaction with SIRT3 (0.2 μM) in 20 mM HEPES buffer (pH = 8.0) containing 100 μM NAD^+ at 37 °C for 2 h. (B) HPLC analysis of the enzymatic reaction of p-H4K16laNBD probe (10 μM) with recombinant SIRT3 (0.2 μM) in 20 mM HEPES buffer (pH = 8.0) containing 100 μM NAD^+ at 37°C for 2 h. (C) Time-dependent fluorescence measurements of p-H4K16laNBD (10 μM) in the presence of HDAC3 (grey) and SIRT3/NAD^+ (red) (λex = 480 nm; λem = 550 nm). The first-order rate constant k of enzymatic reaction is 0.0102 min^{-1} and 0.0059 min^{-1}.

p-H4K16laAlk

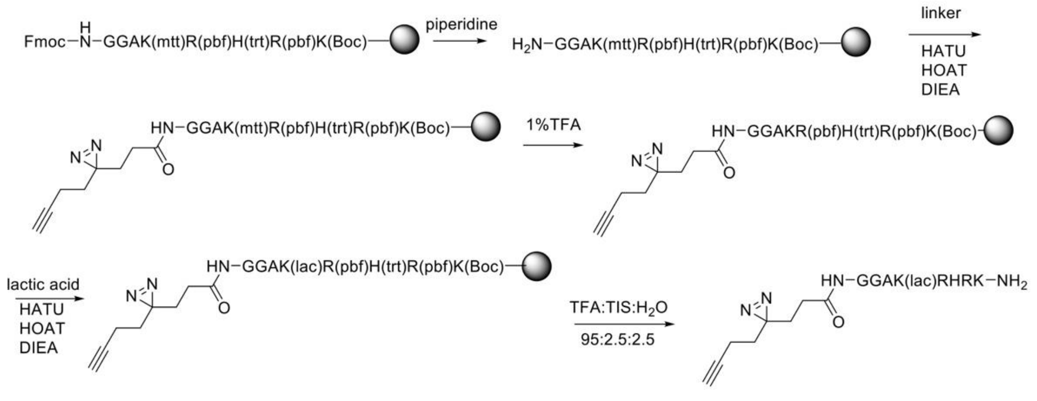

Competitor (H4K16la peptide)

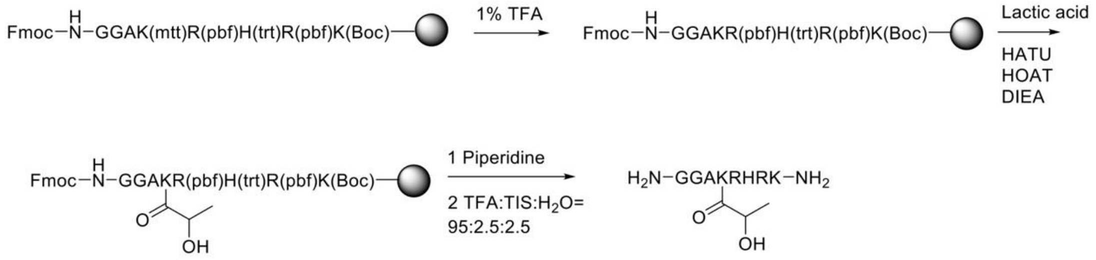

Figure S17. Synthesis procedure of the p-H4K16laAlk (upper) and the competitor (H4K16la peptide) (lower), related to STAR Methods

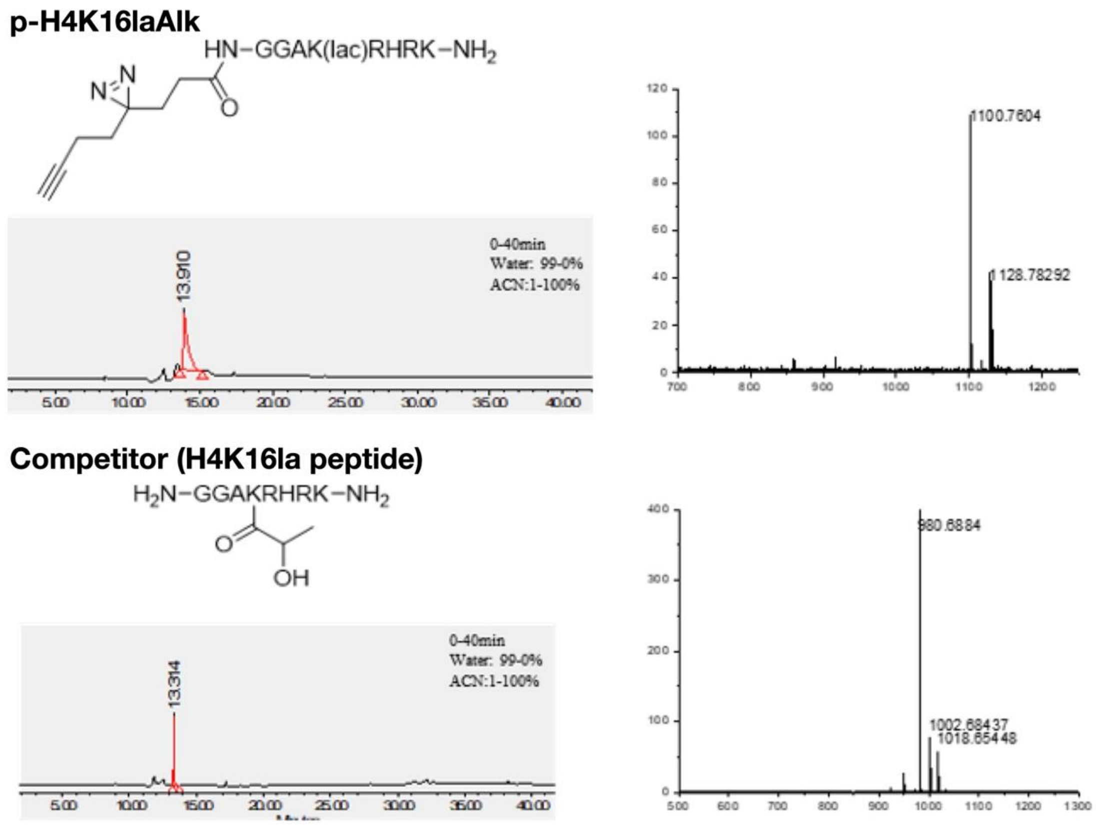

Figure S18. LC-MS analysis of the p-H4K16laAlk (upper) and the competitor (H4K16la peptide) (lower), related to STAR Methods

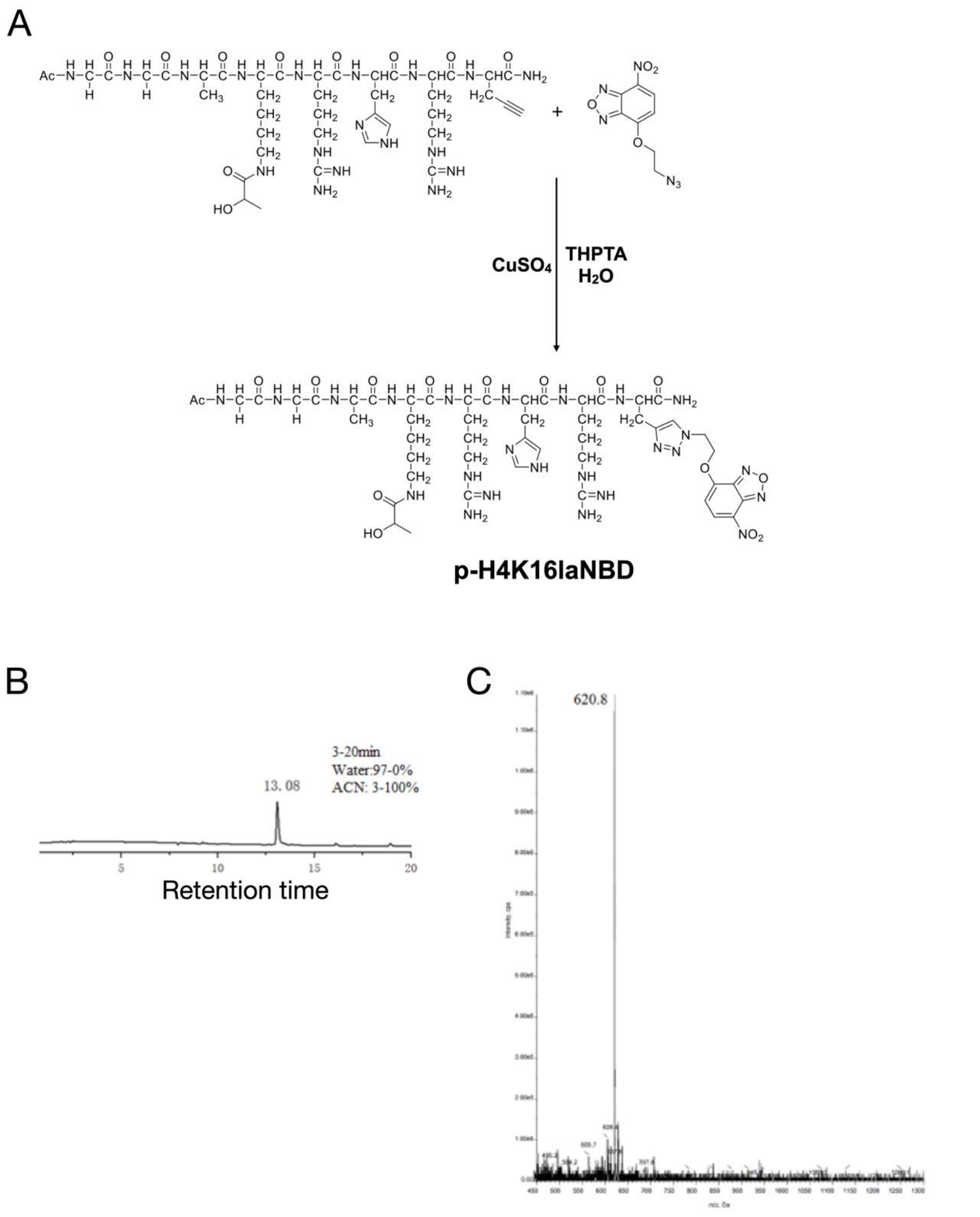

Figure S19. Synthesis procedure and the LC-MS analysis of p-H4K16laNBD, related to STAR Methods

(A) To synthesize p-H4K16laNBD, the peptide sequence Ac-GGAK(lac)RHRPro-NH_2 was synthesized using solid based method. After the purification, the peptide was clicked with N_3-ONBD to give the final probe p-H4K16laNBD.The reaction was conducted for 6 h with strong stirring under the catalyst of Cu(I). (B)(C) The probe was purified and verified by LC-MS.

Table S1. The literature evidence of Sirtuins' preferred sites, related to Figure 1A

Histone site	Sirtuins	Modification types	Description (Sirtuin-histone-acyl-lysine complex structures published from PDB or mentioned in published articles)
H3K9	SIRT1	Ac	SIRT1 binds to H3K9 within the P53 region, and result in decreased H3K9ac level and repressed p53 gene transcription.[1]
	SIRT2	Myr	Published PDB: SIRT2-H3K9myr (4Y6L).[2]
	SIRT3	Pal/Myr/Bhb	Published PDB data: SIRT3-H3K9pal (5BWO) /myr (5BWN)/bhb (5Z93).[3]
	SIRT5	Suc	Published PDB: SIRT5-H3K9suc (3RIY).[4]
	SIRT6	Myr	Published PDB: SIRT6-H3K9myr (3ZG6).[5]
H3K14	SIRT1	Ac	SIRT1 regulate gene expression of HIF-1α via deacetylation on H3K14.[6]
	SIRT2	Ac	Reduced SIRT2 expression caused upregulated H3K14ac level in diabetic cartilage.[7]
	SIRT3	Ac/Bhb	Overexpression of SIRT3 reduced H3K14ac level[8], and SIRT3 is able to de-β-hydroxybutyrylate H3K14 *in vitro.*[9]
H3K56	SIRT1	Ac	SIRT1 binds to H3K56 within the bclaf1 promoter region, and result in decreased H3K56ac level, suppressed bclaf1 expression and inhibited T cell activation.[10]
	SIRT2	Ac	Reduced SIRT2 expression caused upregulated H3K56ac level in diabetic cartilage.[7]
	SIRT3	Ac	Overexpression of SIRT3 reduced H3K56ac level.[8]
	SIRT6	Ac	Deacetylase activity *in vitro.*[11]
H4K16	SIRT1	Ac	SIRT1 represses transcription of TNF-α in THP-1 macrophages via deacetylation on H4K16.[12,13]
	SIRT2	Ac	SIRT2 regulate mitosis via deacetylation on H4K16.[14]
	SIRT3	Bhb	Published PDB: SIRT3-H4K16bhb (5ZGC).[9]
	SIRT5	Ac	SIRT5 deacetylates H4K16ac and regulate the transcription of PDX1.[15]
	SIRT6	Ac	SIRT6 deacetylates H4K16ac and regulate oocyte meiosis.[16]

Table S2. The literature evidence of the nuclear existence of SIRT3, related to Figure 1I, J

SIRT3 type	Cell type	Methods	Conclusion
C-term EGFP labeled SIRT3[9]	HEK293	Colocalization (Z-stack 3D imaging) (DAPI and Anti-EGFP antibody)	C-term EGFP-labeled SIRT3 fluorescence signals in nucleus.
Overexpressed or endogenous SIRT3[9]	HEK293	Cell fractionation study (Western blot))	Overexpressed or endogenous SIRT3 exist in nuclear fractions.
Endogenous SIRT3[9]	HEK293	bhbNa treatment (Western blot))	SIRT3 level increased in chromatin-bound fractions when histone Kbhb level upregulated.
SIRT3 knockdown [17]	Hela	si-RNA knockdown (Western blot)	SIRT3 knockdown caused upregulated pan-Kcr and H3K4cr level
Endogenous SIRT3[17]	Hela	Colocalization (DAPI and anti-SIRT3 N-term antibody)	The full length SIRT3 exists in nucleus.
Endogenous SIRT3[17]	Hela	Cell fractionation study (Western blot)	SIRT3 can be detected in nuclear fractions by using both anti SIRT3 N- and C-term antibodies.
Endogenous SIRT3[18]	HEK293F	Cell fractionation study (Western blot)	SIRT3 (both full length and the processed one lacking N-term 142 amino acids) can be detected in nucleus.
C-term HA tagged SIRT3 (tetracycline induced)[18]	HEK293F	Cell fractionation study (Western blot) and Colocalization (DAPI and anti-SIRT3 N-term antibody)	SIRT3-HA (both full length and the processed one lacking N-term 142 amino acids) can be detected in nucleus.
C-term FLAG tagged SIRT3[19]	HEK293T	Proteomic analysis<br><br>(Identification of SIRT3 interacting proteins)	Lamin B1 (nuclear envelop protein), and KAP1 (heterochromatin-associated protein) were identified by LC-MS/MS.
C-term FLAG tagged SIRT3[19]	HEK293T	Co-IP (Western blot)	SIRT3 interacts with the nuclear proteins, including Lamin B1, KAP1, HP1α, HP1γ, and LBR.
Endogenous SIRT3[19]	hMSCs	Cell fractionation study (Western blot)	Both SIRT3 and Lamin B1 were detected in nuclear fraction.
C-term FLAG tagged SIRT3 overexpressed in SIRT3$^{-/-}$ hMSCs[19]	hMSCs	Colocalization (Z-stack 3D imaging) (Anti Lamin B1 and Anti-FLAG antibody)	SIRT3 and Lamin B1 colocalized at nucleus.

Table S3. Crystal data collection and refinement statistics, related to Figure 2

**Data collection**	**(SIRT3-H3K23la)**	**(SIRT3-H4K16la)**
Wavelength (Å)	0.976	0.976
Resolution range (Å)	42.38 - 1.998 (2.07 - 1.998)	45.24 - 2.501 (2.59 - 2.501)
Space group	$P\ 2_1\ 2_1\ 2_1$	$P\ 6_5$
Unit cell	33.043 84.763 89.187 (Å)<br>90 90 90 (°)	97.449 97.449 53.583 (Å)<br>90 90 120 (°)
Total reflections	25417 (1294)	19276 (1630)
Unique reflections	16948 (1273)	10160 (994)
Multiplicity	1.5 (1.0)	1.9 (1.6)
Completeness (%)	95.93 (72.33)	99.79 (98.61)
Mean I/sigma (I)	15.12 (3.48)	28.75 (4.26)
Wilson B-factor	22.99	33.42
R-merge	0.03378 (0.07989)	0.03693 (0.1884)
R-meas	0.04777 (0.113)	0.05222 (0.2665)
R-pim	0.03378 (0.07989)	0.03693 (0.1884)
CC1/2	0.996 (1)	0.996 (0.843)
CC*	0.999 (1)	0.999 (0.956)
**Refinement**		
Reflections used in Refinement	16948 (1292)	10153 (992)
Reflections used for R-free	1697 (133)	1004 (100)
R-work	0.1570 (0.2149)	0.1877 (0.2366)
R-free	0.2006 (0.2559)	0.2426 (0.3080)
CC (work)	0.954 (0.896)	0.961 (0.844)
CC (free)	0.925 (0.881)	0.938 (0.645)
Number of non-	2370	2281

hydrogen atoms		
macromolecules	2173	2168
ligands	56	6
solvent	169	107
Protein residues	276	274
RMS (bonds)	0.005 (Å)	0.003 (Å)
RMS (angles)	0.78 (º)	0.6 (º)
Ramachandran favored (%)	97.79	97.76
Ramachandran allowed (%)	2.21	2.24
Ramachandran outliers (%)	0.00	0.00
Rotamer outliers (%)	0.00	0.42
Clashscore	2.04	3.67
Average B-factor	27.54	47.05
macromolecules	27.17	46.83
ligands	29.18	39.81
solvent	31.97	51.87
Number of TLS groups	1	1

Statistics for the highest-resolution shell are shown in parentheses.

Table S4. Proteomic analysis: protein identified after enrichment (with enriched ratio≥2, $p < 0.05$), related to Figure 3B

	$Log_2(K_{p\text{-}H4K16laAlk} / K_{p\text{-}H4K16laAlk\ with\ 100\mu M\ competitor})$	-Log(p-value)
**SIRT3**	3.528473	3.880375
**RPL19**	2.936674	1.801582
**DKC1**	2.927341	1.614196
**SRSF1**	2.686776	1.977218
**SRSF7**	2.571431	2.023642
**GMPS**	2.537244	2.094105
**RPL10**	2.473908	1.513292
**RPL18A**	2.468427	1.840031
**EBNA1BP2**	2.413991	2.24365
**TOP1**	2.311893	1.409809
**ARF1**	2.270554	2.947051
**ATAD3A**	2.164889	1.364628
**RPL13**	2.112772	2.172246
**HNRNPH2**	2.035472	1.409859
**RPL7**	1.907576	1.71624
**FBL**	1.894831	2.483103
**RPS3A**	1.84828	1.768023
**TLN1**	1.838418	1.607041
**RPL17**	1.82833	1.472486
**RPL15**	1.770842	1.593327
**CPT1A**	1.75947	4.307828
**RPL3**	1.691245	1.494277
**RPL11**	1.63718	1.557138
**CSNK2A3;CSNK2A1**	1.489653	2.223984
**GTPBP4**	1.453023	1.442969
**RPS6**	1.424533	2.46101
**LMNA**	1.420535	1.937808
**TARS**	1.40882	1.727108
**EIF3A**	1.405017	1.682487
**HP1BP3**	1.390128	1.919034
**CLPB**	1.386087	1.449422
**VDAC3**	1.318909	1.480091
**MYBBP1A**	1.313581	1.768858
**RPS8**	1.3128	1.441611
**ABCD3**	1.31063	1.778466
**RPS11**	1.302059	1.808605
**RPS7**	1.301185	1.770535
**KPNB1**	1.289495	2.431653
**PABPC4**	1.268914	1.421224
**NCLN**	1.262781	2.141539
**SLC25A6;SLC25A4**	1.243965	1.423398
**RPL8**	1.217177	3.47264
**MSN**	1.211431	2.360911

**RFC5**	1.192929	1.934166
**VDAC1**	1.191338	1.404913
**RAB2A;RAB2B**	1.185791	1.61055
**RPL5**	1.138686	1.331613
**NAT10**	1.124737	1.753622
**UQCRC2**	1.115121	1.353112
**RPL18**	1.083732	1.474
**NPM1**	1.051168	1.743963
**CACYBP**	1.043609	1.38192
**HNRNPA1;HNRNPA1L2**	1.036201	2.17814
**PSMD3**	1.032894	1.794367
**ACAT1**	1.008949	2.449278
**RPL4**	1.002317	2.250274

Table S5. Peptide sequences used in NAD+ consumption/cycling assay, related to STAR Methods

Lactyl-lysine sites	Sequences
**H3K9**	QTARK (L/D lactic acid) STGG
**H3K14**	STGGK (L/D lactic acid) APRK
**H3K56**	RRYQK (L/D lactic acid) STEL
**H4K16**	KGGAK (L/D lactic acid) RHRK

Table S6. Peptide sequences used in ITC assay, related to STAR Methods

Lactyl-lysine sites	Sequences
**H3K9**	QTARK (L/D-lactic acid) STGG
**H3K14**	STGGK (L/D-lactic acid) APRK
**H3K56**	RRYQK (L/D-lactic acid) STEL
**H4K16**	KGGAK (L/D-lactic acid) RHRK
**H4K16**	KGGAK (L-lactic acid) RHRK
**H4K16**	KGGAK (D-lactic acid) RHRK

Table S7. Peptide sequences used in HPLC-MS analysis, related to STAR Methods

Lactyl-lysine sites	Sequences
**H3K9**	WQTARK (L/D-lactic acid) STGGW
**H3K18**	WKAPPK (L/D-lactic acid) QLATKW
**H3K23**	WKQLATK (L/D-lactic acid) AARKW
**H4K12**	WKGLGK (L/D-lactic acid) GGAKW
**H4K16**	WKGGAK (L/D-lactic acid) RHRKW

Reference

1. Wang, R.-H., Zheng, Y., Kim, H.-S., Xu, X., Cao, L., Lahusen, T., Lee, M.-H., Xiao, C., Vassilopoulos, A., and Chen, W. (2008). Interplay among BRCA1, SIRT1, and Survivin during BRCA1-associated tumorigenesis. Molecular cell *32*, 11-20.
2. Feldman, J.L., Dittenhafer-Reed, K.E., Kudo, N., Thelen, J.N., Ito, A., Yoshida, M., and Denu, J.M. (2015). Kinetic and structural basis for acyl-group selectivity and NAD+ dependence in sirtuin-catalyzed deacylation. Biochemistry *54*, 3037-3050.
3. Gai, W., Li, H., Jiang, H., Long, Y., and Liu, D. (2016). Crystal structures of SIRT 3 reveal that the α2-α3 loop and α3-helix affect the interaction with long-chain acyl lysine. FEBS letters *590*, 3019-3028.
4. Du, J., Zhou, Y., Su, X., Yu, J.J., Khan, S., Jiang, H., Kim, J., Woo, J., Kim, J.H., and Choi, B.H. (2011). Sirt5 is a NAD-dependent protein lysine demalonylase and desuccinylase. Science *334*, 806-809.
5. Jiang, H., Khan, S., Wang, Y., Charron, G., He, B., Sebastian, C., Du, J., Kim, R., Ge, E., and Mostoslavsky, R. (2013). SIRT6 regulates TNF-α secretion through hydrolysis of long-chain fatty acyl lysine. Nature *496*, 110-113.
6. Dong, S.-Y., Guo, Y.-J., Feng, Y., Cui, X.-X., Kuo, S.-H., Liu, T., and Wu, Y.-C. (2016). The epigenetic regulation of HIF-1α by SIRT1 in MPP+ treated SH-SY5Y cells. Biochemical and biophysical research communications *470*, 453-459.
7. Qu, Z., Ma, X., Huang, S., Hao, X., Li, D., Feng, K., and Wang, W. (2020). SIRT2 inhibits oxidative stress and inflammatory response in diabetic osteoarthritis. Eur Rev Med Pharmacol Sci *24*, 2855-2864.
8. Palomer, X., Román-Azcona, M.S., Pizarro-Delgado, J., Planavila, A., Villarroya, F., Valenzuela-Alcaraz, B., Crispi, F., Sepúlveda-Martínez, Á., Miguel-Escalada, I., and Ferrer, J. (2020). SIRT3-mediated inhibition of FOS through histone H3 deacetylation prevents cardiac fibrosis and inflammation. Signal transduction and targeted therapy *5*, 1-10.
9. Zhang, X., Cao, R., Niu, J., Yang, S., Ma, H., Zhao, S., and Li, H. (2019). Molecular basis for hierarchical histone de-β-hydroxybutyrylation by SIRT3. Cell discovery *5*, 1-15.
10. Kong, S., Kim, S.-J., Sandal, B., Lee, S.-M., Gao, B., Zhang, D.D., and Fang, D. (2011). The type III histone deacetylase Sirt1 protein suppresses p300-mediated histone H3 lysine 56 acetylation at Bclaf1 promoter to inhibit T cell activation. Journal of Biological Chemistry *286*, 16967-16975.
11. Kokkonen, P., Rahnasto-Rilla, M., Mellini, P., Jarho, E., Lahtela-Kakkonen, M., and Kokkola, T. (2014). Studying SIRT6 regulation using H3K56 based substrate and small molecules. European Journal of Pharmaceutical Sciences *63*, 71-76.
12. Chen, G.D., Yu, W.D., and Chen, X.P. (2016). SirT1 activator represses the transcription of TNF-α in THP-1 cells of a sepsis model via deacetylation of H4K16. Molecular Medicine Reports *14*, 5544-5550.
13. Hajji, N., Wallenborg, K., Vlachos, P., Füllgrabe, J., Hermanson, O., and Joseph, B. (2010). Opposing effects of hMOF and SIRT1 on H4K16 acetylation and the sensitivity to the topoisomerase II inhibitor etoposide. Oncogene *29*, 2192-2204.
14. Vaquero, A., Scher, M.B., Lee, D.H., Sutton, A., Cheng, H.-L., Alt, F.W., Serrano, L., Sternglanz, R., and Reinberg, D. (2006). SirT2 is a histone deacetylase with preference for

histone H4 Lys 16 during mitosis. Genes & development *20*, 1256-1261.

15. Ma, Y., and Fei, X. (2018). SIRT5 regulates pancreatic β-cell proliferation and insulin secretion in type 2 diabetes. Experimental and therapeutic medicine *16*, 1417-1425.

16. Han, L., Ge, J., Zhang, L., Ma, R., Hou, X., Li, B., Moley, K., and Wang, Q. (2015). Sirt6 depletion causes spindle defects and chromosome misalignment during meiosis of mouse oocyte. Scientific reports *5*, 1-10.

17. Xiucong, B., Yi, W., Xin, L., Xiao-Meng, L., Zheng, L., Tangpo, Y., Fat, W.C., Jiangwen, Z., Quan, H., and David, L.X. (2014). Identification of 'erasers' for lysine crotonylated histone marks using a chemical proteomics approach. eLife,3,(2014-10-07) *3*.

18. Scher, Michael, B., Vaquero, Alejandro, Reinberg, and Danny (2007). SirT3 is a nuclear NAD⁺-dependent histone deacetylase that translocates to the mitochondria upon cellular stress. Genes & Development.

19. Diao, Z., Ji, Q., Wu, Z., Zhang, W., Cai, Y., Wang, Z., Hu, J., Liu, Z., Wang, Q., and Bi, S. (2021). SIRT3 consolidates heterochromatin and counteracts senescence. Nucleic Acids Research.
